# Randomized controlled trial on the effects of a supervised high intensity exercise program in patients with a hematologic malignancy treated with autologous stem cell transplantation: Results from the EXIST study

**DOI:** 10.1371/journal.pone.0181313

**Published:** 2017-07-20

**Authors:** Saskia Persoon, Mai J. M. ChinAPaw, Laurien M. Buffart, Roberto D. K. Liu, Pierre Wijermans, Harry R. Koene, Monique C. Minnema, Pieternella J. Lugtenburg, Erik W. A. Marijt, Johannes Brug, Frans Nollet, Marie José Kersten

**Affiliations:** 1 Department of Rehabilitation, Academic Medical Center, University of Amsterdam, Amsterdam, The Netherlands; 2 VU University Medical Center, Department of Public and Occupational Health and the EMGO+ Institute for Health and Care Research, Amsterdam, The Netherlands; 3 VU University Medical Center, Department of Epidemiology and Biostatistics and the EMGO+ Institute for Health and Care Research, Amsterdam, The Netherlands; 4 Department of Hematology, Academic Medical Center, University of Amsterdam, Amsterdam, The Netherlands; 5 Department of Hematology, Haga Teaching Hospital, The Hague, The Netherlands; 6 Department of Internal Medicine, St. Antonius Hospital, Nieuwegein, The Netherlands; 7 Department of Hematology, UMC Utrecht, Utrecht, The Netherlands; 8 Department of Hematology, Erasmus MC Cancer Institute, Rotterdam, The Netherlands; 9 Department of Hematology, Leiden University Medical Center, Leiden, The Netherlands; Hospital Universitario de Salamanca, SPAIN

## Abstract

**Background:**

This single blind, multicenter randomized controlled trial aimed to evaluate the effectiveness of a supervised high intensity exercise program on physical fitness and fatigue in patients with multiple myeloma or lymphoma recently treated with autologous stem cell transplantation.

**Methods:**

109 patients were randomly assigned to the 18-week exercise intervention or the usual care control group. The primary outcomes included physical fitness (VO_2peak_ and W_peak_ determined using a cardiopulmonary exercise test; grip strength and the 30s chair stand test) and fatigue (Multidimensional Fatigue Inventory) and were assessed prior to randomization and after completion of the intervention or at similar time points for the control group. Multivariable multilevel linear regression analyses were performed to assess intervention effects.

**Results:**

Patients in the intervention group attended 86% of the prescribed exercise sessions. Of the patients in the control group, 47% reported ≥10 physiotherapy sessions, which most likely included supervised exercise, suggesting a high rate of contamination. Median improvements in physical fitness ranged between 16 and 25% in the intervention group and between 12 and 19% in the control group. Fatigue decreased in both groups. There were no significant differences between the intervention and control group.

**Conclusion:**

We found no significant beneficial effects of the supervised high intensity exercise program on physical fitness and fatigue when compared to usual care. We hypothesized that the lack of significant intervention effects may relate to suboptimal timing of intervention delivery, contamination in the control group and/or suboptimal compliance to the prescribed exercise intervention.

**Trial registration:**

Netherlands Trial Register—NTR2341.

## Introduction

High dose chemotherapy followed by autologous stem cell transplantation (auto-SCT) improves the outcome of patients with multiple myeloma or lymphoma [1]. Nevertheless, this treatment may negatively impact the patient’s health-related quality of life (HRQoL) [2, 3]. Among Dutch patients, the most frequently reported long-term difficulties were problems with physical fitness and fatigue [4]. An effective additional treatment for these problems could significantly improve the HRQoL of patients treated with auto-SCT.

Exercise may improve physical fitness and reduce cancer-related fatigue among patients with cancer [5–8], including patients treated with a stem cell transplantation for a hematologic malignancy [9–11]. However, a part of the previous studies may have underestimated the efficacy of exercise interventions as two of the established principles of exercise training, overload and progression, were not always adequately addressed in the design of these exercise interventions [9, 12, 13]. The overload principle states that the training stimuli must be larger than those the individual is accustomed to. Progression denotes that the program should be progressive and continuously provide adequate overload over time [12–14].

In addition to a small pilot RCT (n = 19) [15], the effectiveness of exercise interventions delivered predominantly or completely after hospitalization for SCT as compared with usual care has been studied in two larger randomized controlled trials (RCTs), with slightly different results: Hacker et al. [16] compared a resistance exercise intervention with a usual care plus attention control with health education intervention in 67 patients, and found positive effects of the exercise program on fatigue and functional ability, and no significant group x time interaction effect for physical activity, physical fatigue, muscle strength and quality of life [16]. In contrast, in 131 patients, Knols et al. [17] found significant beneficial effects of a supervised resistance and aerobic exercise program on functional exercise capacity, lower extremity muscle strength, and emotional functioning, but not on fatigue, overall QoL and physical, role and social functioning [17]. Both studies also included patients after allogeneic SCT and neither used the gold standard cardiorespiratory exercise test to assess cardiorespiratory fitness. Because there are considerable differences in treatment and associated symptoms between patients with different types of hematologic malignancies and because of the differences between allogeneic and auto-SCT [1], it is important to examine intervention effects in a relatively homogeneous group of patients.

The primary aim of the EXercise Intervention after Stem cell Transplantation (EXIST) study was therefore to determine the effectiveness of an individualized high intensity supervised exercise program on physical fitness (i.e. cardiorespiratory and muscular fitness) and fatigue in a relatively homogeneous sample of patients with multiple myeloma or lymphoma recently treated with auto-SCT. Secondary outcomes were body composition, HRQoL, distress and physical activity.

## Materials and methods

The EXIST study was a multicenter, prospective, single blind RCT. The study was registered at the Netherlands Trial Register (NTR2341; 27 may 2010) and approved by the Medical Ethics Committee of the Academic Medical Center (AMC; 25 June 2010) and by the boards of the Antoni van Leeuwenhoek Hospital (Amsterdam), St. Antonius Hospital (Nieuwegein), Haga Teaching Hospital (Den Haag), University Medical Center (Utrecht), Isala (Zwolle), Erasmus MC/Daniel den Hoed (Rotterdam), VU University Medical Center (Amsterdam) and Leiden University Medical Center (Leiden). The trial protocol and the CONSORT checklist are available as supplementary information (S1 Protocol and S1 Checklist) and details of the study design have been published previously [18, 19]. The authors certify that all ongoing and related trials for this intervention have been registered.

Patients were recruited between March 2011 and February 2014. Patients treated with auto-SCT for multiple myeloma or lymphoma were eligible for the study 6–14 weeks after transplantation if they had sufficiently recovered (Hb>10.5 g/dL, platelets>80x10^9/^l), and were able to undergo exercise testing, and participate in an exercise intervention. In case patients received consolidation chemotherapy or radiation therapy following auto-SCT, patients were included 2–6 weeks after completion of this treatment. All participants gave written informed consent. At baseline and prior to randomization, a sports physician screened the patients to confirm eligibility.

Patients were randomly assigned to the exercise group or to the usual care control group. Randomization was concealed, took place after completion of baseline assessments (T0) and was performed by an independent data manager using a validated software program (TENALEA Clinical Trial Data Management System; Netherlands Cancer Institute, Amsterdam, the Netherlands). Randomization was stratified by transplant center and diagnosis and proceeded using block randomization with block sizes varying randomly between 2, 4 and 6. The first author (SP) was blinded to allocation and assessed the study outcomes (except for body composition, which was assessed by the sports physician) and performed the analyses.

### Exercise intervention and usual care

Patients in the intervention group followed an 18-week high-intensity resistance exercise and interval training program [19–21], and followed five counseling sessions aiming to improve compliance with the exercise program and motivate them to pursue an active lifestyle outside the exercise program. The intervention took place in local physiotherapy practices and was supervised by instructed physiotherapists. Exercise sessions took place twice a week in the first 12 weeks and once a week from week 13 onwards. The counseling sessions lasting 5–15 minutes each were scheduled in week 1, 4, 10, 12 and 18.

The resistance exercises consisted of four standardized exercises (vertical row, leg press, bench/chest press and pull over/flies) and two additional exercises for the abdominal muscles and the upper legs. In week 1–12, two sets of 10 repetitions were performed at 65–80% of the indirectly determined one repetition maximum (1-RM). Hereafter, the number of repetitions increased to 20 per set and the resistance decreased to 35–40% of the 1-RM. The interval training consisted of two times eight minutes of cycling. The maximal short exercise capacity (MSEC) assessed with the steep ramp test [22] was used to determine the adequate exercise intensity of the interval bouts. In week 1–8, blocks of 30s at 65% MSEC were alternated with blocks of 60s at 30% MSEC. Henceforth, the latter block was shortened to 30s. To ensure adequate training progression, both the indirect 1-RM measurements and the steep ramp test were performed every four weeks and the training load was adjusted accordingly [19–21].

Patients in the control group were not specifically motivated by the members of the study team to exercise or participate in sports, physiotherapy or rehabilitation programs, but they were not restricted in their physical activities or in the use of health care services.

### Measurements

Physical tests were performed either at the AMC or at the Sports Medical Advice Center Rotterdam, and questionnaires were filled out at home. Follow-up measures (T1) were performed directly after completion of the intervention program or at a similar time point for the control group. Between baseline and follow-up, patients filled out cost-diaries in which the number of attended physiotherapy sessions and medication use were recorded. The physiotherapists reported about session attendance and adverse events in a training log. Sociodemographic variables were assessed by self-report and baseline clinical characteristics were extracted from medical records.

#### Session attendance and contamination

Session attendance was determined as the number of exercise sessions attended between T0 and T1. Information about contamination was collected from the reports from the sports physician and the patients’ cost-diaries. Contamination was defined as the attendance of ≥10 physiotherapy sessions for patients in the control group.

#### Primary outcome measures

Assessments of physical fitness included cardiorespiratory and muscular fitness. Cardiorespiratory fitness was assessed using a cardiopulmonary exercise test, performed on a cycle ergometer (Lode Excalibur, Groningen, the Netherlands). Outcomes were the highest continuous 15s interval values for oxygen uptake (VO_2peak_; MasterScreen CPX, CareFusion, Hoechberg, Germany) and the highest achieved workload (W_peak_).

Grip strength of the dominant hand was measured with a grip strength dynamometer (Hydraulic Hand Dynamometer, North Coast Medical Inc., Morgan Hill, USA) [23]. The highest score of three attempts was used in the analyses. Lower body functional performance was tested using the 30s chair stand test [24].

Fatigue was measured using the Multidimensional Fatigue Inventory (MFI) [25]. The MFI has five subscales (general, physical and mental fatigue, reduced activity and reduced motivation) on which patients could score between 4 and 20.

#### Secondary outcome measures

Secondary outcomes included the body mass index (BMI), sum of four skinfolds, maximal isometric voluntary torque of the m. quadriceps (Biodex System 3, New York, USA; added to the protocol), HRQoL (functional scales and global QoL scale of the European Organisation Research and Treatment of Cancer—Quality of Life Questionnaire Core 30, EORTC-QLQ-C30 [26], and symptom scales of the EORTC Myeloma Module, QLQ-MY20) [27], distress (Hospital Anxiety and Depression Scale, HADS) [28, 29], self-reported physical activity (Physical Activity Scale for the Elderly, PASE) [30] and objectively measured physical activity by accelerometers (Actitrainer, Actigraph, Fort Walton Beach, USA) worn on five consecutive days during waking hours. The epoch time was set on 60s. Data were processed using ActiLife Software version 6.10.2 (ActiGraph, Pensacola, Florida, USA). Vertical accelerations were converted in counts per minute (cpm) and non-wear time was defined as >90 consecutive minutes of zero counts [31]. Data of patients with at least 4 days with ≥8 hours valid wear time were included in the analyses.

### Power calculations

Power calculations were conducted according to the standard sample-size calculation for trials with two study arms presented by Twisk [32]. We expected group differences of 7.5 ml/kg/min (SD = 7) in VO_2peak_; 0.2 kg (SD = 0.1) in grip strength and 3.5 (SD = 4) points in fatigue (MFI) [20, 21, 33], and consequently needed 42 patients per group to assess between-group differences. We originally aimed to include 120 patients, but as our dropout of 15% was smaller than the 30% which was anticipated, the study was closed for accrual after including 109 patients.

### Statistical analyses

After checking the assumptions, we conducted multivariable multilevel linear regression analyses with a two level structure (individual; transplant center) to detect between-group differences in outcome measures at post-test. The intervention was regressed on the post-test value of the outcome, adjusted for baseline levels, age, gender and education level. Data were analyzed on an intention-to-treat basis. In addition, we also analyzed the data using multiple imputation techniques. We had one or more missing values in 17% of the cases for the patient reported outcomes, in 46% of the cases in one or more physical fitness outcomes, and in 58% of the cases in the physical fitness outcomes when combined with accelerometry data. The high percentage of cases for the physical fitness outcomes was mainly due to missings in the VO_2_peak (36%, of which 15% was due to loss to follow-up). The percentage missing on the W_peak_ was 23%. We used univariate logistic regression analyses to determine if the missings on our primary outcomes measures were selective. VO_2peak_ values were more likely to be missing for patients assessed at the Sports Medical Advice Center Rotterdam than for patients assessed at the AMC (OR = 13.0, 95%CI = 2.7;63.0). Furthermore, patients with a lower education level were more likely to have missing values for the 30s chair stand test (OR = 0.2, 95%CI = 0.04;0.8) and general fatigue (OR = 0.1, 95%CI = 0.01;0.9). Assuming these associations to be random, we imputed missing data based on the missing at random assumption, by creating 20, 45 or 60 datasets, depending on the proportion missing values [34]. Effect modification by age, gender and diagnosis was checked by adding the interaction term with group allocation to the regression model. Analyses were performed using IBM SPSS statistical software (version 20.0. Armonk, NY: IBM Corp) and test results were considered significant if *p*<0.05.

#### Post hoc analyses

We conducted post hoc analyses to study differences in effects between patients who attended ≥10 sessions of physiotherapy (users: patients in the intervention group and physiotherapy users in the control group) and patients who attended <10 sessions (non-users). Physiotherapy attendance was regressed on the post-test value of the outcome, adjusted for baseline levels, age, gender and education level.

## Results

**Fig 1:**
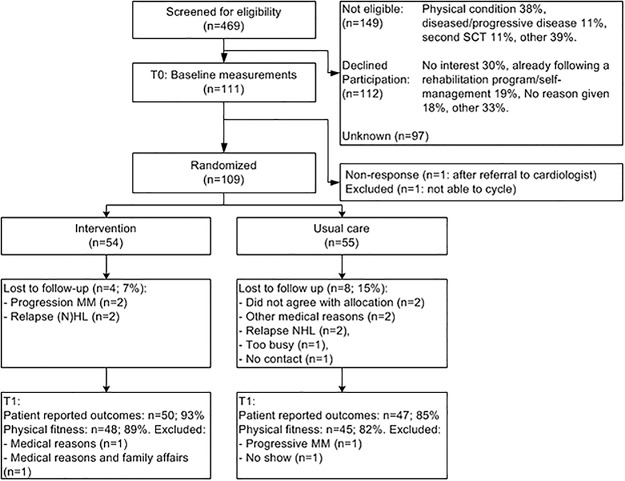
Consort diagram.

Between March 2011 and February 2014, 469 patients were screened for eligibility of whom 150 (32%) were not eligible, 113 (24%) declined to participate, 97 (21%) did not participate for unknown reasons, and 109 patients (23%) participated (Fig 1). The median age of the participants was 55 years (range 19–67; Table 1). One patient in the control group received radiation treatment during the study, and at follow-up eight patients (16%) in the intervention and six patients (13%) in the control group received maintenance therapy. Except for the larger proportion of patients with a high education level in the control group (44 vs 28%), the baseline characteristics were well balanced.

**Table 1:** Baseline demographics and clinical characteristics of participants in the exercise intervention and usual care control group.

	Exercise Intervention group (n = 54)	Usual care control group (n = 55)
Gender, male, n (%)	32 (59)	37 (67)
Age, median(range), years	53.5 (20–67)	56 (19–67)
Married/Living together, n (%)	45 (83)	46 (84)
Higher education level, n (%)	15 (28)	24 (44)
Sports history, yes^a^, n (%)	33 (61)	36 (66)
Cancer type, n (%)		
MM	29 (54)	29 (53)
(N)HL	25 (46)	26 (47)
Time since auto-SCT, median (range), days	75 (42–275)	72 (40–233)
Remission status after auto-SCT, n (%)		
CR	37 (69)	32 (58)
VGPR/PR	16 (30)	22 (40)
SD/PD	1 (2)	1 (2)
Comorbidity	2 (0–6)	2 (0–5)
Neuropathy, n (%)	14 (26)	18 (33)
Musculoskeletal disorders, n (%)	38 (70)	28 (51)
Cardiovascular disease or risk factors		
Risk factors, n (%)	11 (20)	12 (22)
Other, n (%)	12(22)	15 (27)
Respiratory disease, n (%)	7 (13)	7 (12)
Other, n (%)	13 (24)	8 (13)

Abbreviations: MM: multiple myeloma; (N)HL: (non-)Hodgkin lymphoma; auto-SCT: autologous stem cell transplantation; CR: Complete response; VGPR: Very good partial response; PR: partial response/remission; SD: Stable disease; PD: progressive disease

^a^ participating in sports at least once a week before diagnoses/relapse.

The last follow-up measurement was performed in July 2014. Twelve patients were lost to follow-up (Fig 1) and four additional patients did not visit a test center at follow-up. Consequently data of 97 patients (89%) were available for the patient reported outcomes and data of 93 patients (85%) for the physical fitness assessments. Time between baseline and follow-up was similar in both groups. In case of delays and/or interruptions of the exercise program or due to the study logistics, it was possible that missed exercise session(s) were rescheduled after T1.

During the study, eight serious adverse events were reported, four in each group. None of these events were considered to be related to study participation. One patient in the intervention group strained his calf muscles during a training session, but recovered from this injury within the intervention period.

### Session attendance and contamination

Patients in the intervention group attended on average 25.8 (SD = 3.8) of the prescribed 30 exercise sessions and 36 patients (75%) attended ≥80% of the sessions. Main reasons for non-attendance were: illness or injuries (34%), holidays (27%) and session took place after T1 (19%). Of the patients in the control group, 22 (47%) reported to have used ≥10 sessions of physiotherapy. It is likely that these sessions included supervised exercise, but we were unable to retrieve detailed information about the content of these sessions.

### Intervention effects on primary and secondary outcomes

In both groups, cardiorespiratory and muscular fitness improved and fatigue decreased during the study period (Table 2; S1 Fig). Median improvements in physical fitness ranged from 16 to 25% in the intervention group and from 12 to 19% in the control group. General and physical fatigue declined with a median of 25% and 32% in the intervention group and 12% and 25% in the control group, respectively. There were no significant between-group differences in any of the outcomes. The imputation of missing data did not change conclusions (data not shown). Age was a significant effect modifier in the effect on objectively measured physical activity, with older patients having a smaller increase (β_interaction_ = -5.1, 95%CI = -9.0;-1.3).

**Table 2:** Effects of the intervention on primary and secondary outcome measures in patients treated with auto-SCT.

	Exercise intervention group (n = 50)^±^		Usual care control group (n = 47)^±^		β (95%CI)^#^
	T0	T1	T0	T1	
***Primary outcome measures***					
**Cardiorespiratory fitness**					
VO_2peak_ (mL/kg/min)^a^	21.7(4.8)	26.0 (6.3)	21.2 (5.4)	24.2 (6.6)	1.2 (-0.5;2.9)
W_peak_ (Watt/kg)^b^	2.0(0.5)	2.4 (0.7)	2.0 (0.6)	2.4 (0.8)	0.1 (-0.1;0.2)
**Muscular fitness**					
Chair stand test (number)^c^	15.5 (4.6)	18.7 (6.0)	14.5 (4.6)	17.1 (4.3)	0.7 (-0.5;1.9)
Grip strength (kg)	35.5 (10.7)	40.9 (12.0)	36.9 (10.1)	41.3 (11.7)	1.3 (-0.5;3.1)
**Fatigue**					
Physical fatigue^d^	13.2 (4.2)	9.8 (4.4)	14.4 (4.8)	11.1 (5.0)	-0.8 (-2.2;0.7)
General fatigue^e^	12.7 (3.8)	10.0 (4.5)	13.5 (4.3)	11.8 (4.8)	-1.4 (-2.9;0.1)
Mental fatigue^d^	10.0 (4.3)	9.7 (4.5)	10.3 (4.8)	9.7 (4.2)	0.1 (-1.4;1.6)
Reduced activity^d^	12.2 (4.1)	9.6 (3.9)	13.6 (4.5)	10.8 (4.7)	-0.4 (-1.7;0.8)
Reduced motivation^d^	8.8 (3.8)	8.0 (3.2)	9.9 (3.8)	8.4 (3.3)	0.1 (-1.0;1.2)
***Secondary outcome measures***					
**Body composition**					
BMI (kg/m^2^)	25.6 (4.6)	26.3 (4.7)	25.1 (3.6)	25.5 (3.8)	0.2 (-0.3;0.7)
Sum skinfolds (mm)^f^	61.5 (22.8)	64.5 (25.1)	60.8 (24.6)	64.0 (25.5)	-0.4 (-5.0;4.2)
**Muscular fitness**					
Maximal torque m. quadriceps (Nm)^g^^+^	145.6 (51.4)	173.9 (55.9)	143.9 (52.3)	165.3 (57.2)	6.6 (-4.4;17.6)
**Physical activity**					
Accelerometry (cpm)^h^	189.0 (79.2)	241.4 (92.3)	197.2 (103.5)	250.6 (135.0)	-9.8 (-50.1;30.5)
PASE-score^i^	96.9 (99.5)	131.3 (82.4)	77.0 (59.3)	113.5 (97.9)	1.9 (-30.3;34.1)
**Health related quality of life**					
Global quality of life	62.2 (18.0)	75.0 (18.7)	62.2 (20.6)	73.4 (18.4)	2.3 (-4.1;8.6)
Physical functioning	74.5 (18.3)	83.1 (19.1)	74.6 (16.9)	84.1 (15.3)	-2.3 (-6.6;2.0)
Role functioning	60.0 (31.6)	81.0 (23.8)	62.8 (29.7)	73.4 (31.8)	9.7 (-0.1;19.5)
Emotional functioning	83.2 (14.9)	86.2 (16.3)	81.4 (19.1)	86.0 (14.7)	-1.1 (-6.2;3.9)
Cognitive functioning	87.3 (15.6)	83.7 (18.0)	80.5 (21.8)	82.3 (20.4)	-2.7 (-8.7;3.4)
Social functioning	71.7 (27.2)	86.0 (20.3)	74.1 (25.0)	83.7 (20.4)	2.5 (-5.1;10.0)
Disease symptoms^d^^‡^	16.6 (13.1)	18.7 (12.8)	19.8 (14.6)	18.8 (17.1)	0.8 (-4.6;6.3)
Side effects^d^^‡^	21.5 (9.7)	10.6 (8.1)	21.9 (11.8)	12.1 (8.9)	-0.5 (-4.7;3.7)
**Distress**					
Anxiety	4.4 (3.5)	4.1 (4.1)	4.3 (3.3)	3.9 (2.5)	0.02 (-1.0;1.0)
Depression^d^	3.8 (3.8)	3.2 (3.4)	4.1 (3.6)	2.9 (3.0)	0.4 (-0.6;1.3)

Data are mean (±SD).

Abbreviation BMI: Body Mass Index; PASE: Physical Activity Scale for the Elderly.

^±^Physical fitness assessments: exercise intervention group (n = 48), usual care control group (n = 45).

^#^Adjusted for age, gender, education level and including random intercept.

^+^Patients assessed at the AMC only.

^‡^Patients with multiple myeloma only.

Missing data due to

^a^technical problems (n = 8), leakage of/took off face mask/inadequate performance (n = 8), cardiovascular problems (n = 4), change in beta-blockers prescription (n = 2), thrombocytopenia (n = 1);

^b^cardiovascular problems/change in beta-blocker prescription (n = 4), inadequate performance (n = 4), thrombocytopenia (n = 1);

^c^back injury (n = 2);

^d^incomplete questionnaire (n = 1);

^e^incomplete questionnaire (n = 2);

^f^not performed (n = 6);

^g^test not yet included in protocol (n = 3), back injury (n = 2), inadequate performance (n = 1), thrombocytopenia (n = 1), technical problems (n = 1);

^h^insufficient wear time (n = 16), technical problems (n = 6), initialization/download failure (n = 4).

^i^incomplete questionnaire (n = 5).

A higher score for physical activity and functioning scales indicated a higher physical activity level and better HRQoL, respectively. A higher score on fatigue, symptom scales and distress indicated worse HRQoL and higher levels of fatigue and distress, respectively.

### Post hoc analyses

Baseline demographic and clinical characteristics of the users and non-users were well balanced. Physiotherapy users had a larger increase in self-reported physical activity level (β = 35.0, 95%CI = 0.9;69.1), but a smaller reduction in anxiety (β = 1.3, 95%CI = 0.2;2.3) and depression (β = 1.5, 95%CI = 0.5;2.5) at follow-up than non-users (Table 3).

**Table 3:** Effects of ≥10 physiotherapy sessions on primary and secondary outcome measures in patients treated with auto-SCT.

	Users (n = 72)^±^		Non-users (n = 25) ^±^		β (95%CI)^#^
	T0	T1	T0	T1	
***Primary outcome measures***					
**Cardiorespiratory fitness**					
VO_2peak_ (mL/kg/min)^a^	21.0 (4.8)	25.2 (6.0)	22.8 (5.8)	25.0 (7.9)	1.8 (-0.1;3,8)
W_peak_ (Watt/kg)^b^	1.9 (0.5)	2.4 (0.7)	2.0 (0.6)	2.4 (0.8)	0.1 (-0.04;0.2)
**Muscular fitness**					
Chair stand test (number)^c^	15.3 (4.5)	18.2 (5.6)	14.4 (5.0)	17.1 (4.2)	0.3 (-1.0;1.7)
Grip strength (kg)	35.7 (10.4)	40.7 (11.5)	37.6 (10.2)	42.3 (12.9)	0.5 (-1.5;2.6)
**Fatigue**					
Physical fatigue^d^	13.8 (4.5)	10.8 (4.8)	13.8 (4.6)	9.5 (4.4)	0.8 (-0.8;2.4)
General fatigue^e^	13.2 (4.0)	11.1 (4.9)	12.8 (4.3)	10.1 (3.9)	0.5 (-1.1;2.2)
Mental fatigue^d^	10.3 (4.6)	9.9 (4.5)	9.9 (4.3)	9.2 (3.7)	0.5 (-1.1;2.2)
Reduced activity^d^	12.8 (4.4)	10.4 (4.4)	13.3 (4.2)	9.7 (4.1)	0.9 (-0.5;2.3)
Reduced motivation^d^	9.1 (3.8)	8.3 (3.2)	9.9 (4.0)	8.0 (3.5)	0.5 (-0.7;1.8)
***Secondary outcome measures***					
**Body composition**					
BMI (kg/m^2^)	25.3 (4.6)	25.9 (4.7)	25.6 (2.7)	26.1 (3.1)	0.1 (-0.5;0.6)
Sum skinfolds (mm)^f^	60.9 (24.7)	64.3 (26.5)	61.9 (20.4)	64.0 (21.1)	0.9 (-4.2;6.0)
**Muscular fitness**					
Maximal torque m. quadriceps (Nm)^g^^+^	140.8 (46.6)	167.2 (51.2)	156.9 (64.5)	177.2 (71.3)	3.9 (-8.5;16.3)
**Physical activity**					
Accelerometry (cpm)^h^	191.8 (81.7)	238.6 (87.3)	196.8 (116.6)	265.8 (169.8)	-23.1 (-67.3;21.1)
PASE-score^i^	89.1 (88.6)	132.6 (94.1)	81.1 (62.2)	94.9 (74.7)	35.0 (0.9;69.1)*
**Health related quality of life**					
Global quality of life	61.2 (19.1)	71.9 (20.1)	65.0 (19.5)	81.0 (10.6)	-6.5 (-13.5;0.4)
Physical functioning	73.6 (17.1)	82.2 (18.0)	77.3 (18.9)	87.7 (14.6)	-2.6 (-7.4;2.1)
Role functioning	57.2 (31.4)	75.9 (27.4)	73.3 (25.0)	81.3 (30.2)	2.8 (-8.5;14.1)
Emotional functioning	83.1 (16.8)	85.3 (16.1)	80.0 (17.5)	88.3 (13.4)	-5.3 (-10.8;0.2)
Cognitive functioning	84.3 (19.6)	81.9 (20.5)	83.3 (18.0)	86.0 (14.2)	-4.9 (-11.5;1.7)
Social functioning	70.8 (26.2)	83.1 (21.9)	78.7 (25.2)	90.0 (13.6)	-4.1 (-12.5;4.3)
Disease symptoms^d^^‡^	16.9 (12.4)	18.3 (13.4)	21.0 (16.8)	19.8 (18.5)	0.6 (-5.2;6.5)
Side effects^d^^‡^	21.6 (9.7)	10.7 (7.5)	21.9 (13.2)	13.0 (10.5)	-1.8 (-6.3;2.6)
**Distress**					
Anxiety	4.3 (3.4)	4.3 (3.8)	4.7 (3.5)	3.2 (1.8)	1.3 (0.2;2.3)*
Depression^d^	3.9 (3.8)	3.4 (3.5)	4.2 (3.2)	2.1 (1.4)	1.5 (0.5;2.5)*

Data are mean (±SD).

Abbreviation: BMI: Body Mass Index; PASE: Physical Activity Scale for the Elderly

*p<0.05.

^±^Physical fitness assessments: users (n = 69), non-users (n = 24).

^#^Adjusted for age, gender, education level and including random intercept.

^+^Patients assessed at the AMC only.

^‡^Patients with multiple myeloma only.

Missing data due to

^a^technical problems (n = 8), leakage of/took off face mask/inadequate performance (n = 8), cardiovascular problems (n = 4), change in beta-blockers prescription (n = 2), thrombocytopenia (n = 1);

^b^cardiovascular problems/change in beta-blockers prescription (n = 4), inadequate performance (n = 4), thrombocytopenia (n = 1);

^c^back injury (n = 2);

^d^incomplete questionnaire (n = 1);

^e^incomplete questionnaire (n = 2);

^f^not performed (n = 6);

^g^test not yet included in protocol (n = 3), back injury (n = 2), inadequate performance (n = 1), thrombocytopenia (n = 1), technical problems (n = 1);

^h^insufficient wear time (n = 16), technical problems (n = 6), initialization/download failure (n = 4).

^i^incomplete questionnaire (n = 5).

A higher score for physical activity and functioning scales indicated a higher physical activity level and better HRQoL, respectively. A higher score on fatigue, symptom scales and distress indicated worse HRQoL and higher levels of fatigue and distress, respectively.

## Discussion

In this single blind, multicenter RCT in patients recently treated with auto-SCT we found no significant favorable effects of a supervised high intensity exercise program on physical fitness, fatigue, body composition, HRQoL, distress or physical activity compared to usual care.

The lack of significant intervention effects on fatigue, global QoL and physical, role and social functioning is in line with findings from the study of Knols et al. [17] who evaluated the effectiveness of a 12-week supervised resistance and aerobic exercise program among patients after allogeneic or auto-SCT. However, in contrast to our study, they found significant improvements on lower extremity muscle strength and functional exercise capacity [17]. In addition, the Resistance and Endurance exercise After ChemoTherapy (REACT) study, in which the effects of a comparable exercise intervention were evaluated, found significant beneficial effects on cardiorespiratory fitness and fatigue among 277 cancer survivors who completed neo-adjuvant or adjuvant chemotherapy [35]. Interestingly, the absolute changes in cardiorespiratory fitness and general and physical fatigue in the REACT study were similar to our results. We hypothesize that the lack of significant between-group differences in our study may be related to suboptimal timing of intervention delivery, contamination in the control group, and/or suboptimal compliance to the prescribed exercise intervention.

In our study and in the study by Knols et al. [17] fatigue and HRQoL improved in both the intervention and control group. The lack of significant between-group differences suggests that exercise does not speed up natural recovery directly after (auto-)SCT. A previous systematic review concluded that exercise during hospitalization had beneficial effects on fatigue and HRQoL at discharge from the hospital after allogeneic SCT [11]. Therefore, further studies are needed to clarify the optimal timing of intervention delivery.

In addition, contamination in our study may have diluted the effects of exercise. We performed post-hoc analyses to assess potential favorable effects of ≥10 sessions of physiotherapy, regardless of treatment group. Physiotherapy users had a higher self-reported physical activity level but smaller declines in levels of anxiety and depression at follow-up than non-users. Possibly, physiotherapy users in the control group had more complaints and/or higher care needs during the study for which they sought help. For most outcomes, it is unclear whether the physiotherapy sessions prevented further deterioration or had no beneficial effects.

The contamination in the control group can be explained by the rapid increase in attention for cancer rehabilitation in the Netherlands in the last decade and the clinical guideline that was published in 2011 [36]. Consequently, physiotherapy practices specialized in the treatment of cancer survivors and specialized group-based cancer rehabilitation programs are now widely available and the costs of treatment are often (partly) reimbursed by the health insurance. Notably, Kuehl et al. [37] recently reported a similar contamination rate (54%) among German allogeneic SCT survivors who were allocated to the control arm (muscle relaxation program) of an exercise RCT, although the definitions that were used differed (attendance of ≥10 physiotherapy sessions versus self-reported amount of sport activity at 180 days after SCT). We agree with Kuehl et al. that the risk of contamination should be taken into account in exercise trials [37].

Session attendance was good, but detailed information on the dose received (frequency, intensity, time and type of the exercises completed) has not been analysed yet. Performing a process evaluation can help illustrating the actual implementation of the intervention and may thus help to explain our study results [38, 39].

Despite the lack of significant intervention effects on physical fitness, supervised exercise interventions may still be valuable in a specific subgroup of patients. Given the necessity of sufficient levels of physical fitness to perform the activities of daily living [40, 41], improving physical fitness remains important in patients with low levels of physical fitness after auto-SCT and in those who perceive physical (e.g. co-morbidities) or psychological (e.g. fear) barriers to exercise on their own.

Strengths of this study are the inclusion of a large and relatively homogenous group of patients treated with auto-SCT, the use of well accepted and gold standard outcome measures and the solid design and methodology, including blinding of outcome assessment and statistical analyses. Furthermore, the intervention program was individualized and based on established exercise principles. Our attrition rate of 15% was slightly below the mean attrition rate of 18% reported in a previous review on attrition and adherence in studies evaluating exercise in patients treated with hematopoietic SCT [42]. However, we had a relatively high number of missing values, mainly in the VO_2_peak (36%), and the results for this outcome measure should be interpreted carefully. The previously mentioned contamination can be considered another limitation of our study. Besides, although the response rate was at least 34% and comparable to the response rate of the REACT study (37%) [35], only 23% of the screened patients actually participated in the study. As we also have no information on demographic and clinical characteristics of the patients who declined participation, caution is warranted when generalizing the results of our study to all patients treated with an auto-SCT.

In conclusion, in this well-designed RCT among 109 patients treated with auto-SCT, we found no significant beneficial effects of the high intensity supervised exercise program on physical fitness, fatigue and HRQoL when compared to usual care. The lack of significant intervention effects may be explained by suboptimal timing of intervention delivery, by suboptimal compliance to the prescribed exercise dose and/or by contamination in the control group.

## Supporting information

S1 Protocol Study protocol. (PDF)

S1 Checklist CONSORT checklist. (DOC)

S1 Fig Effects of the intervention on VO_2peak_, handgrip strength and general fatigue. (TIF)

S1 File Anonymized dataset. (SAV)
