# Supplementary material for: Randomized controlled trial on the effects of a supervised high intensity exercise program in patients with a hematologic malignancy treated with autologous stem cell transplantation: Results from the EXIST study
Source: PLoS One. 2017 Jul 20;12(7):e0181313. doi: 10.1371/journal.pone.0181313 (PMC5519072; doi:10.1371/journal.pone.0181313)
Supplement: S1 Protocol — (PDF) [file pone.0181313.s001.pdf]

Physical exercise to improve fitness and combat fatigue in patients with multiple myeloma and (non-) Hodgkin's lymphoma treated with high dose chemotherapy and autologous stem cell transplantation

Study protocol

Principal investigators:
Dr. M.J. Kersten,
Academic Medical Center
Dept of Hematology, F4-224
Phone: + 31-20-5665785
Fax: +31-20-6919743
m.j.kersten@amc.nl

Dr. M.J.M. Chin A Paw
VU University Medical Center
EMGO Institute for Health and Care Research
Dept of Public and Occupational Health
Phone: +31-20-4448203
m.chinapaw@vumc.nl

Prof. dr. F. Nollet
Academic Medical Center
Dept of Rehabilitation
Phone: +31-20-5664049
f.nollet@amc.nl

Prof. dr. J. Brug
VU University Medical Center
EMGO Institute for Health and Care Research
Dept of epidemiology & biostatistics
Phone: +31-20-4448180
j.brug@vumc.nl

Study coordinator:
S. Persoon, MSc
Academic Medical Center
Dept of Rehabilitation
Phone: + 31-20-5668387
s.persoon@amc.nl

Registration:
Academic Medical Center
Dept of Hematology F4-224
M. Spiering
Phone: +31-20-5669111 beeper 59255
Fax: +31-20-6919743
m.spiering@amc.nl

First version: 12 April 2010
Final version: 12 April 2010
Amendement-1: 23 September 2010
Amendement-2: 11 May 2011

Amendement-3: 26 September 2011
Amendement-4: 12 December 2011
Amendement-5: 6 June 2012
Amendement-6: 1 May 2013
Amendement 7: 11 June 2013
Date of activation: 10 December 2010
Approved: 25 June 2010

1. Scheme of the study

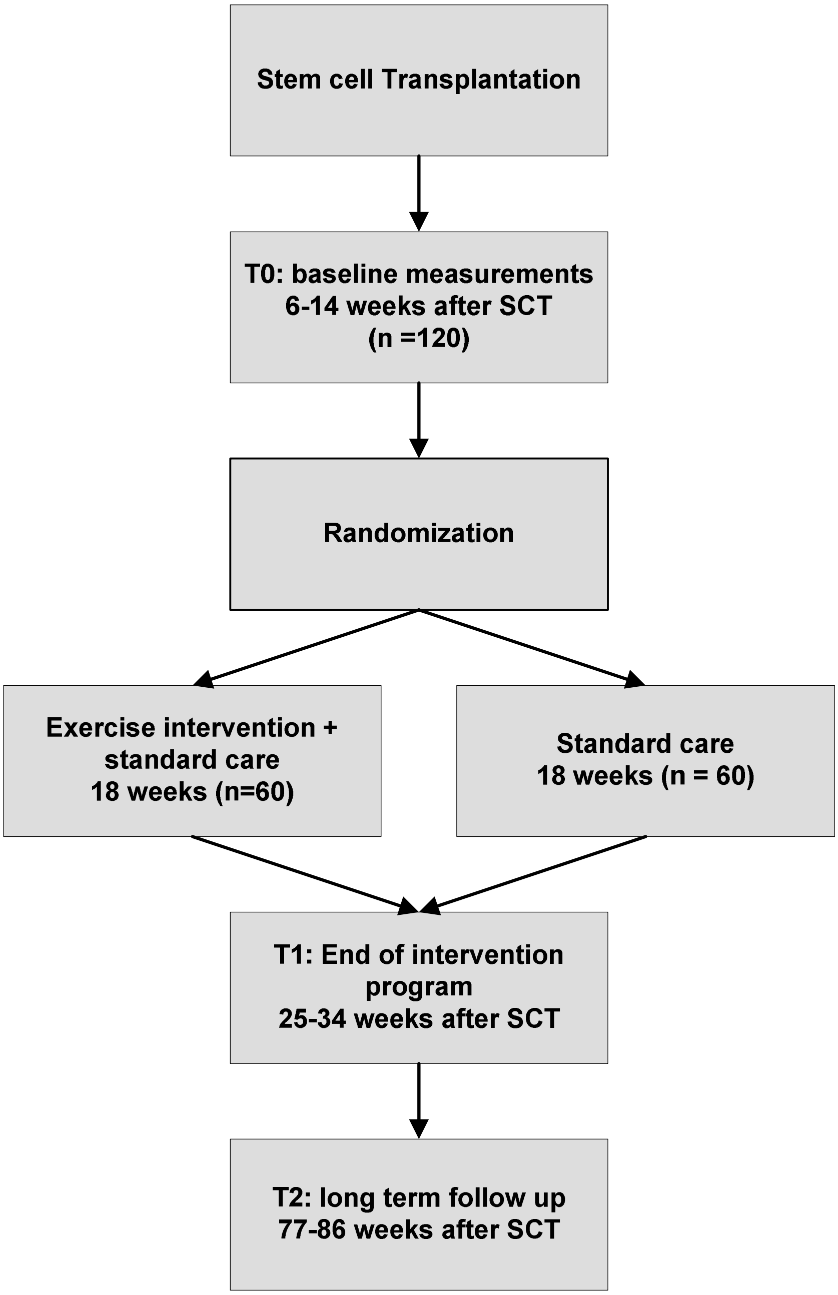

2. Table of contents

1. Scheme of the study 3

2. Table of contents 4

3. List of investigators 6

4. Summary 7

5. Background 8
5.1 Exercise interventions in the literature 8
5.2 Preliminary results 9
    5.2.1 Exercise intervention program 9
    5.2.2 Focus group interviews. 10
5.3 Economic evaluation 10
5.4 Conclusion 10
5.5 Hypothesis 10

6. Study Objectives 11

7. Study design 12
7.1 Pilot study 12
7.2 Randomized controlled trial 12
7.3 Inclusion and exclusion criteria 12
7.4 Recruitment 12
7.5 Randomization & blinding 13

8. Treatment 14
8.1 Intervention arm 14
    8.1.1 Resistance exercise 14
    8.1.2 Interval training 14
    8.1.3 Counseling 14
8.2 Standard care (control arm) 15
8.3 End of protocol treatment 15
8.4 Timing and location of the investigations 16
8.5 Required assessments at baseline, after treatment and during follow up 16

9. Outcome measures 18
9.1 Primary outcome measures: 18
9.2 Secondary outcome measures: 19
9.3 Other measurements 20
9.4 Monitoring of compliance and attrition 21

10. Safety reporting 22
10.1 Adverse event (AE) 22
10.2 Serious adverse event (SAE) 22
    10.2.1 Unexpected SAE 22
10.2 Protocol treatment period 22
10.3 Reporting of serious adverse events 22

11. Statistical considerations 24
11.1 Sample size and statistical power calculations 24
11.2 Statistical analysis 24
    11.2.1 Baseline characteristics 24
    11.2.2 Primary analysis 24

11.2.3 Economic evaluation 25

12. Ethical and regulatory standards 26
12.1. Ethical principles 26
12.2. Laws and regulations 26
12.3. Informed Consent 26
12.4. Ethical review 26
12.5. Confidentiality 26
12.6 Compensation for injury 26
13.1 Data monitoring 27
13.2 Annual progress report 27
13.3 End of study report 27
13.4 Public disclosure and publication policy 27

14. Glossary of abbreviations 28

15. References 29

3. List of investigators

Principal investigators:

Dr. M.J. Kersten,
Academic Medical Center
Dept of Hematology, F4-224
Meibergdreef 9
1105AZ Amsterdam
Phone: + 31-20-5665785
Fax: +31-20-6919743
m.j.kersten@amc.uva.nl

Prof. dr. F. Nollet
Academic Medical Center
Dept of Rehabilitation, A01-425
Meibergdreef 9
1105AZ Amsterdam
Phone: +31-20-5664049
f.nollet@amc.uva.nl

Dr. M.J.M. Chin A Paw
VU University Medical Center
EMGO Institute for Health and Care Research
Dept of Public and Occupational Health
Van der Boechorststraat 7
1081 BT Amsterdam
Phone: +31-20-4448203
m.chinapaw@vumc.nl

Prof. dr. J. Brug
VU University Medical Center
EMGO Institute for Health and Care Research
Dept of Epidemiology & Biostatistics
Van der Boechorststraat 7
1081 BT Amsterdam
Phone: +31-20-4448180
j.brug@vumc.nl

Study coordinator:

S. Persoon, MSc
Academic Medical Center, Amsterdam
Dept of Rehabilitation
Meibergdreef 9
1105 AZ Amsterdam
Phone: + 31-20-5668387
s.persoon@amc.uva.nl

Local investigators:

Dr. H.R. Koene
St. Antonius ziekenhuis, Nieuwegein
Dept of Internal Medicine
h.koene@antoniusziekenhuis.nl

Dr. P. Wijermans
Haga ziekenhuis, Den Haag
Dept of Hematology
p.wijermans@hagaziekenhuis.nl

Dr. J.W. Baars
NKI-AVL, Amsterdam
Dept of Internal Medicine
j.baars@nki.nl

Dr. M.C.Minnema
UMC Utrecht
Dept of Hematology
m.c.minnema@umcutrecht.nl

dr.P. Lugtenburg
Erasmus MC
Meander Medisch Centrum
Dept of Hematology
p.lugtenburg@erasmusmc.nl

dr. Kersting
Leiden University Medical Center
Dept of Hematology
s.kersting@lumc.nl

dr. O. Visser
VU University Medical Center
Dept of Hematology
o.visser@vumc.nl

dr. M. van Marwijk Kooy
Isala Klinieken
Dept of Medical Oncology
m.van.marwijk@isala.nl

4. Summary

Rationale: The use of high-dose chemotherapy (HDC) and autologous stem cell transplantation (SCT) has improved the outcome of hematologic malignancies such as multiple myeloma and (non-) Hodgkin's lymphoma. However, the long term side effects of this treatment can have a strong negative impact on quality of life. Patients often complain of severe and persistent fatigue and are compromised in their ability to perform normal physical activities. Physical exercise interventions after SCT can have positive effects on physical fitness, quality of life and fatigue. However, the trials conducted so far were of poor to moderate quality, with methological shortcomings related to trial design, sample size, choice of comparison groups, outcome measures and duration of follow up. There is a need for a rigorous, appropriately controlled assessment of the effectiveness and cost-effectiveness of exercise in these patients.

Objective: (1) To evaluate the effectiveness of an individualized exercise program in comparison to standard care with respect to fatigue, physical fitness and health-related quality of life in patients with hematologic malignancies who have undergone HDC and autologous SCT. (2) To evaluate the cost-effectiveness of this exercise program.

Study design: (1) Multicenter, prospective, single blind randomized controlled trial.

Study population: Patients (≥ 18 years) with multiple myeloma or (non-)Hodgkin's lymphoma who have undergone induction chemotherapy followed by HDC and autologous SCT.

Intervention: The patients (n=120) will be randomized to one of two groups: (1) exercise intervention + standard care (n=60) or (2) standard care (n=60).

Main study parameters: At baseline, at completion of the intervention and at 12 months follow-up cardiopulmonary fitness, muscular strength, fatigue and secondary outcome measures will be assessed.

5. Background

The use of high-dose chemotherapy (HDC) and autologous stem cell transplantation (SCT) has improved the outcome of hematologic malignancies such as multiple myeloma (MM) and (non-) Hodgkin's lymphoma (NHL/HL). It has become standard of care in these diseases in first line and at relapse respectively. However, the long term side effects of this treatment can have a strong negative impact on quality of life [1]. Up to 75% of patients interviewed at least twelve months post-SCT report severe and persistent fatigue, and are compromised in their ability to engage in normal physical activities [2] About 30% is not able to return to work during the course of the first two years after treatment [3]. Physical deconditioning, resulting from diminished cardiovascular and pulmonary function, reduced muscular strength and cachexia were also highly prevalent [4]. With increasing survival rates, attention should not only be focused on improving cure rates but also on improving recovery after treatment, thereby increasing long-term quality of life. Preliminatory results show that exercise interventions can counter the physical deconditioning after SCT, and thus might also improve the quality of life in these patients.

5.1 Exercise interventions in the literature

There are a few trials that focused specifically on the effects of physical exercise in patients with hematologic malignancies treated with HDT and SCT. These trials suggested that exercise intervention programs during or after HDT and SCT can improve physical performance, quality of life and fatigue status [see for example 5,6,7,8,9,10,11,12]. Systematic reviews of Liu et al., 2009 and Wiskemann & Huber, 2008 also found encouraging results of exercise intervention studies in these patients. Among these results were an increase in physical fitness, health-related quality of life (HRQL) and psychological well-being. No unexpected or negative effects were encountered. Despite these good results, the systematic reviews conclude that further research is necessary [13,14].
In the study of Liu et al., 2009, only three of the eight reviewed exercise intervention studies were randomized controlled studies. The overall quality of many studies reviewed was limited, with shortcomings related to trial design, sample size, choice of comparison groups, outcome measures and duration of follow up [13]. Wiskermann & Huber support these conclusions [14]. These reviews show that there is a need for well designed, randomized clinical trials using a well defined study population, an appropriate control group and valid and reliable outcome measures to confirm the findings of these trials and to facilitate evidence baced therapy.

The trials performed thus far were also very heterogeneous in terms of the type of interventions used, i.e. aerobic exercise versus strength versus mixed-type, moderate versus high intensity exercise, and home-based versus clinically supervised training. Most of them have focused on isolated aerobic exercise during or after the stem cell transplantation. Strength exercise programs and combined intervention strategies have been employed rarely [13,14]. This is somewhat surprising since muscle atrophy is a common problem in cancer patients [15,16]. Normally, muscle atrophy results from a sedentary lifestyle. In cancer patients, muscle atrophy can also be induced by the effects of tumor factors and the side-effects of medication (e.g. glucocorticoids or chemotherapeutic agents [4,17,18]) on skeletal muscle structure and function. These effects are even more pronounced in patients undergoing HDC and SCT, because of the nature of the drugs being used (among others high dose steroids and neurotoxic agents), and because of the morbidity associated with the neutropenic phase after SCT (fever, infections, mucositis, malnutrition) often leading to prolonged bed rest. Considerable evidence suggests that the ability to perform physical tasks in daily life is determined by a threshold level of muscular strength [19]. It is therefore important that exercise interventions aim to minimize muscle atrophy or even stimulate muscle hypertrophy.

Skeletal muscle has shown great adaptability with appropriate training stimuli even in cases of severe muscle atrophy and fatigue [4]. Progressive strength exercise has been shown to increase lean body mass, muscle protein mass and contractile force, and improve physical function in healthy young and elderly subjects [20]. As a consequence, strength exercise in cancer patients seems to be a potent physiological intervention for regaining lost muscle mass and improving muscle quality, ultimately resulting in improved physical fitness, reduced fatigue and an improved overall quality of life [21,22].
Considering the usefulness of both resistance and endurance exercise, an exercise program that combines these two exercise types seems optimal. An example of such a program is the high intensity strength and interval endurance training program developed at the Maxima Medical Center (MMC) in Veldhoven. This study builds on the preliminary results of this exercise program.

5.2 Preliminary results

5.2.1 Exercise intervention program

In 2008, de Backer et al. developed an 18 week high intensity strength and interval training program for application after chemotherapy. Fifty-seven patients (age 24 to 73 years) who had received chemotherapy for lymphomas, breast, gynaecologic, testicular, or colorectal cancer completed the program [23,24]. Outcome measures were changes in muscular strength, maximal aerobic capacity ($VO_{2\ max}$), body composition (using DXA) and HRQL. The high-intensity strength exercise program was well tolerated by all patients. The adherence rate was 84% (6 patients dropped out because of cancer recurrence, and 5 patients because of other reasons such as personal reasons, not interested anymore, disease of the spouse, malaise). Significant improvements (30 to 105%) in muscle strength were found with effect sizes varying from 1.32 to 2.68. VO2 max increased significantly by 10% in men and by 13% in women, with an average absolute increase of 3.5 ml/kg/min. Improved levels of $VO_{2\ max}$ may reduce the strain of daily activities, and consequently may allow for more moderate intensity activities such as household activities and walking. Different functional scales of HRQL improved ($p<0.01$), with effect sizes varying from 0.47 to 0.82. Fatigue diminished significantly after exercise by 50% with an effect size of -0.83 ($p<0.01$). Muscle strength correlated significantly with physical functioning before and after the exercise program.

Since this study lacked a control group, 49 patients who followed the high intensity strength training program were compared with a historical control group of 22 patients treated with chemotherapy in the same period but not participating in a rehabilitation program [24]. Compared with the historical control group, muscle strength at one year follow-up was significantly higher in trained patients. Hierarchical multiple regression showed that muscle strength accounted for an additional 24% ($F=19.07$; $p<0.001$) of the variance in physical functioning. Based on these results it was concluded that a supervised, high-intensity strength and interval training program has persistent and substantial beneficial effects on muscle strength, maximal aerobic capacity, and HRQL in cancer survivors after treatment.

Although not included in a formal study, dr. Goof Schep at MMC has in the last few years trained 8 patients who had undergone a stem cell transplantation because of a hematologic malignancy. All 8 patients completed the exercise program and rated it very positively. There were no adverse effects. Compared to the whole group of cancer patients trained at MMC, the patients after SCT had a very low $VO_{2\ max}$ at the start of the program (range 31-115% of the Jones norm, median 60%; the average in healthy persons being 130%), and required more individualized training.

The high intensity strength and interval training program developed at MMC is promising with respect to rehabilitation of cancer patients, but needs to be further explored and tested in the group of SCT patients. This will be done in this multicenter, randomized controlled trial (RCT).

5.2.2 Focus group interviews.

We performed focus group interviews with patients having undergone autologous SCT for MM, HL or NHL in the AMC. Out of 13 patients interviewed, 12 were interested in participating in an exercise-based rehabilitation trial, provided that they would be able to train close to their home. All 12 patients preferred out-of-home supervised training. After explanation of the assessment procedures used in the proposed RCT, all 12 patients had no objections to undergo such testing.

5.3 Economic evaluation

Several studies have shown that patients who have been treated with high dose chemotherapy and SCT use a lot of resources [25-27], and are often not able to return to work [28-31]. To our knowledge, there are currently no data on the cost-effectiveness of exercise intervention programs in these patients. It is therefore important to study whether the high-intensity exercise intervention, is cost-effective in comparison with standard care

5.4 Conclusion

The question which will be answered in the currently proposed study is whether high intensity strength and interval training based on the program by de Backer et al. [23,24] is effective in patients who have undergone high dose chemotherapy and autologous SCT. More specifically, the effects on cardiopulmonary function, muscular strength and fatigue will be evaluated. Furthermore, the cost-effectiveness of the program will be determined.

5.5 Hypothesis

We hypothesize that following the exercise program and standard care will lead to

1. improved physical fitness;
2. lower levels of fatigue;
3. less mood disturbance, higher levels of daily activities, and improved HRQL;
4. a higher partial and full return to work rate when compared to standard care only;
5. will be cost-effective when compared to standard care.

6. Study Objectives

Primary objective:

- To evaluate the effectiveness of a state-of-the-art individualized high-intensity exercise program in comparison to standard care with respect to fatigue, physical fitness, and HRQoL in patients with hematologic malignancies who have undergone HDT and autologous SCT.

Secondary objective:

- To evaluate the cost-effectiveness of this exercise program in comparison to standard care.

7. Study design

7.1 Pilot study

The intervention in this study is based on the program that has been developed and tested at MMC [32,33]. Prior to the trial a pilot study in 5 SCT patients will be performed to evaluate the feasibility, and if necessary fine tuning of the intervention and to test the study logistics. At the end of the program the patient will be interviewed about: (1) the perceived efficacy of and satisfaction with the intervention program and (2) need for changes to the program.

7.2 Randomized controlled trial

This multicenter, prospective, single blind, RCT will compare an exercise intervention and standard care with standard care only. The 120 patients participating in the study will be randomized to either the intervention arm (n = 60) or the control arm (n = 60). In addition to the standard care, patients in the intervention arm will take part in an 18-week individualized supervised high-intensity exercise program. This program will start 6-14 weeks after SCT. Patients in the control group are treated according to standard care.

7.3 Inclusion and exclusion criteria

Inclusion criteria

- Patients diagnosed with MM in first line or with HL/NHL in first line or first relapse, who have undergone HDC and autologous SCT 6 to 12 weeks ago or who where recently (< 8 months) treated with HDC and autologous SCT and who have completed radiotherapy or their last cycle of consolidation chemotherapy 2 to 6 weeks ago..
- Sufficiently recovered from the SCT and having peripheral blood recovery;
- Age ≥ 18 years
- Ability to cycle on a bicycle ergometer against a load of at least 25 Watt;
- Ability to walk at least 100 meters independently without crutches/cains or walking frame;
- Written informed consent.

Exclusion criteria

- Tandem autologous-allogeneic SCT;
- Severe cognitive impairment;
- Severe emotional instability;
- Insufficient mastery of the Dutch language;
- Presence of extensive osteolytic lesions with risk of fracture;
- Serious cardiopulmonary and cardiovascular conditions;
- Other disabling comorbidity interfering with the intervention program or influencing outcome parameters (a.o. having a pacemaker , epileptic seizures and poorly regulated diabetes mellitus);
- Severe infections;
- Relapse/progression of disease.

7.4 Recruitment

Patients will be recruited at the Academic Medical Center (Amsterdam), Antoni van Leeuwenhoek hospital (Amsterdam), St. Antonius Hospital (Nieuwegein), Haga Ziekenhuis (Den Haag), Onze Lieve Vrouwe Gasthuis (Amsterdam), Erasmus MC (Rotterdam), University Medical Center (Utrecht), Isala klinieken (Zwolle), VU University Medical Center (Amsterdam) and Leiden University Medical Center. All potentially eligible patients will be identified and informed of the study by their treating physician. The patients will receive written information of the study to take home.

7.5 Randomization & blinding

Randomization will be stratified according to transplant center and diagnosis. Eligible patients will be randomized to one of the two arms: (1) exercise intervention and standard care or (2) standard care. Randomisation will be done using the software package ALEA developed by TENALEA [32].

Outcomes will be assessed by blinded and independent trained professionals. At the beginning of the assessment patients will be instructed not to reveal their group allocation.

8. Treatment

8.1 Intervention arm

Besides the standard care (see paragraph 8.2) patients in the intervention arm follow an 18-week exercise program. This program consists of high-intensity resistance and interval training. The patients will train on specialized resistance training equipment and on bicycle ergometers. Training takes place in a local physical therapy practices supervised by physical therapists. Furthermore, the physical therapist will counsel the patient to maintain an active lifestyle. A detailed training manual will be available for the physical therapist. The structure of the intervention program is shown in table 1.

<u>8.1.1 Resistance exercise</u>

The resistance program consists of six exercises targeting the large muscle groups as follows:

1. vertical row (focusing on longissimus, biceps brachii, rhomboideus)
2. leg press (quadriceps, glutei, gastrocnemius)
3. bench press (pectoralis major, triceps)
4. pull over (pectoralis, triceps brachii, deltoideus, trapezius);
5. abdominal crunch (rectus abdominis);
6. lunge (quadriceps, glutei, hamstrings).

In some cases, one of these exercises can be replaced by an alternative exercise (for instance when the patient is not able to perform the exercises or when the appropriate exercise machine is not available in the physical therapy practice). The alternatives are described in the detailed manual.
Indirect one repetition maximum (1-RM) measurements will be performed every 4 weeks. The 1-RM is the greatest resistance that can be moved through the full range of motion in a controlled manner with good posture and this measurement will be used to guide the training: In the first 12 weeks, resistance exercises are performed at 65 to 80% of 1-RM and consist of two sets of 10 repetitions. From week 12 onwards, the emphasis shifts from muscle strength to muscle endurance involving exercise with less resistance (35-40% of 1-RM) but more (20) repetitions.

<u>8.1.2 Interval training</u>

Interval training consists of cycling two times eight minutes, before and after the resistance exercises. The steep ramp test is performed every 4 weeks to determine the right resistance. The subject is instructed to cycle at a speed between 70 and 80 revolutions per minute (RPM), starting at a work rate of 25 Watt for 30 seconds. Hereafter the load is increased by 25 Watts every 10 seconds. The test ends if cycling speed falls below 60 RPM and the maximal short exercise capacity (the maximal workload; MSEC) is recorded. Results of the steep ramp test were described in a previous publication [32]. In the first eight weeks, those eight minutes consist of alternating 30 seconds at 65% of the MSEC and 60 seconds at 30%. From week nine onwards those eight minutes consist of alternating 30 seconds at 65% and 30 seconds at 30% of the MSEC.

<u>8.1.3 Counseling</u>

A behavioural motivation component is included to improve compliance and to stimulate physical activity outside the exercise program in addition to and after completion of the exercise program. From week 12 onwards patients are encouraged to meet the recommendations made by the American College of Sports Medicine and the American Heart Association [33]. Specific program elements include the provision of general and motivational information, both verbally and via folders, about physical activity and the desired intensity of activity based on the Borg Scale rating perceived exertion [34]. The physiotherapist uses basic counselling techniques and instruction sheets.

Table 1. Structure of the exercise program

Week	Type of training	Number of training sessions	Aim of the training
1-12	Resistance training and interval training (2 x 8 minutes).	2x per week, 60 minutes.	1. improve coordination and muscle hypertrophy and hereby improving muscle force.<br>2. become familiar with exercise program.<br>3. increasing aerobic capacity.<br>4. overcoming the fear of physical activity.<br>5. increasing the pleasure in being physical active.
13-18	Resistance training and interval training (2 x 8 minutes).	1 x per week, 60 minutes.	1. promote independence.<br>2. maintain muscle force.<br>3. improve muscle endurance.<br>4. improve aerobic capacity.
1, 4, 10, 12, 18, 22	Counseling.	6 x 15 minutes	1. encourage patients to pursue an active lifestyle.<br>2. give information about desired intensity and duration of physical activities.<br>3. fill out the activity diary.
**Training: 30 sessions a 60 minutes = 30 hours**<br>**Counseling: 6 sessions a 15 minutes = 1.5 hours**			

8.2 Standard care (control arm)

Both the patients in the intervention arm and the patients in the control arm receive standard care. The care will vary according to doctors' and patients' preferences and cannot be standardized. However, information on physical activity/training of patients in the control arm will be obtained from the physical activity questionnaire and cost-diaries. In the Netherlands, participation in the '*Herstel&Balans'* program is possible starting at least three months after completing the cancer treatment. This rehabilitation program is based on a bio-psychosocial approach [35,36]. The program takes 3 months, during which groups of 12-16 participants visit the rehabilitation center for group-based physical rehabilitation and psycho-education. The physical rehabilitation is based on the graded activity theory and consists of supervised sessions, twice a week and is being offered at more than 50 locations in the Netherlands. The training will be mostly focused on aerobic exercise and leisure sports rather than on high intensity strength training. Currently, 10-20% of patients after SCT participate in the '*Herstel&Balans'* program. The majority of patients start with this program 6 months or longer after transplantation.

8.3 End of protocol treatment

Reasons for going off protocol treatment are:

- Early relapse;
- Injuries and diseases, (not) related to the assessments or exercise therapy of this study;
- incompliance of the patient;
- Death;
- Completion of protocol.

8.4 Timing and location of the investigations

Figure 1 shows the timing of the assessments. The initial screening before start of the intervention (t=0) is crucial to identify unknown health problems, to guide exercise, analyse the physiological and muscular limitations that need to be improved by training and to determine the physical loading capacity of individual patients in concert with possible physical limitations [23].

Assessment procedures will be performed at the Academic Medical Center Amsterdam or in Rotterdam (patients Erasmus MC only) according to a detailed and standardized protocol. A physician and the researcher perform the measurements (except for the DXA scan). The assessment of cardiopulmonary fitness and muscle strength will take approximately one hour; filling out the questionnaires at t=0, t=1 and t=2 will take 45 minutes (which can be done at home); filling out the cost diaries will take approximately 10-15 minutes each time.

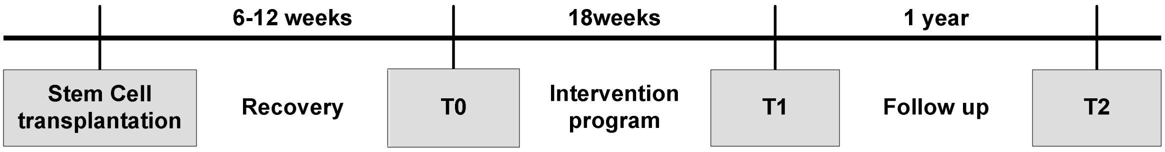

Fig 2. Timeline study. T0: baseline; T1: post treatment; T2: long term follow-up.

8.5 Required assessments at baseline, after treatment and during follow up

Table 2 shows when the different parameters will be assessed and below the table the associated variables or instruments are listed. The measurements are described in the following paragraph.

Table 2. Measurements at different time points

	T0	T1	T2
**Clinical data**			
Medical history[1]	X		
Disease status and treatment[2]	X	X	X
Adverse events	X	X	X
**Physical tests**			
Physical examination[3]	X	X	X
Lab tests[4]			
Cardiopulmonary fitness[5]	X	X	X
Muscular strength[6]	X	X	X
Body composition and bone mineral density[8]	X		X
**Questionnaires**			
Fatigue[7]	X	X	X
Health-related quality of life[9]	X	X	X
Physical activity[10]	X	X	X
Mood disturbance[11]	X	X	X
Functioning in daily life[12]	X	X	X
Return to work[13]	X	X	X
Satisfaction with the intervention[14]		X	
cost questionnaires[15]	Monthly		Every 3 months

1. Date of diagnosis, subtype of disease, stage of disease, history of therapy.
2. Response to treatment, progression or relapse of disease and data on any additional treatment will be recorded from medical records.
3. Height, weight, waist and hip circumferences, four skinfolds (biceps, triceps, suprailiacal and subscapular). At T0 a physician will also look for comorbidities that might interfere with the intervention program or influence outcome.
4. Lab tests: Complete blood count (haemoglobin, platelets, WBC), only if no recent lab values (<3 weeks) are available.
5. $VO_{2\,max}$-test.
6. Maximal handgrip strength, chair stands test and fixed dynamometry.
7. Multidimensional Fatigue Inventory (MFI) and the Fatigue Quality List (FQL) questionnaires
8. DXA-scans.
9. EORTC Quality of Life Questionnaire C30 (EORTC QLQ-C30), the EORTC Myeloma Module (QLQ-MY20), the EORTC Chemotherapy-induced peripheral neuropathy module (QLQ-CIPN20) and the EuroQol (EQ5D).
10. PASE Questionnaire and 5-day recordings of the Actitrainer accelerometer (Actigraph, Fort Walton Beach Florida, USA). A series of questions will be used to assess pre-illness lifestyle, current attitudes towards and beliefs about exercise in general and exercising after stem cell transplantation and potential predictors of compliance with the exercise program. These questions will be asked at baseline only.
11. Hospital Anxiety and Depression Scale (HADS) questionnaire.
12. Impact on Participation and Autonomy (IPA) Questionnaire.
13. RTW-questionnaire.
14. Satisfaction questionnaire; intervention arm only.
15. Cost questionnaires will be administered on a monthly basis during the period between T0 and T1. Hereafter they will be administered once every 3 months.

9. Outcome measures

9.1 Primary outcome measures:

1. Cardiopulmonary fitness

Cardiopulmonary fitness is related to the ability to perform large muscle, dynamic, moderate-to-high intensity exercise for prolonged periods. Performance of such exercise depends on the functional state of the respiratory, cardiovascular, and skeletal muscle systems. $VO_{2\,max}$ testing is well established and there are international guidelines concerning standardization and interpretation strategies [37]. This technique has been shown feasible in cancer survivors [4]. The $VO_{2\,max}$ test will be performed on a cycle ergometer and a ramp test design will be used. ECG, saturation and blood pressure will be monitored and heart rate (HR) and gas exchange variables will be recorded continuously. The test will be supervised by a physician.

By applying this test a broad range of parameters can be assessed. Among these parameters are the

- Maximal work rate (Wmax)
- Maximal aerobic power ($VO_{2\,max}$)
- Ventilation threshold (VT)
- Maximal HR (HRmax)
- VO_2 at VT (VO_2 VT)
- HR at VT (HR VT)
- Maximal ventilation (V_E max)
- % Saturation of Hb with O_2 (S)
- Maximal respiratory frequency (f max)
- VO_2 at respiratory exchange ratio = 1 (VO_2 RER 1)
- HR at respiratory exchange ratio = 1 (HR RER 1)

Prior to the aerobic exercise test pulmonary function will be assessed at rest by measuring the forced air expiratory volume in 1 second (FEV-1), the forced vital capacity (VC), inspiratory capacity (IC) and the maximal voluntary ventilation (MVV) using a portable spirometer [38].

2. Muscular strength

Muscular strength refers to the external force that can be generated by a specific muscle or muscle group. Upper extremity muscular strength will be assessed using a grip strength dynamometer and lower extremity muscular strength by the '30 s chair stands' test. In addition muscular strength of the m. quadriceps will be measured isometrically using a fixed dynamometer (Biodex System).

Maximal handgrip strength is performed three times for each hand. The mean score attained for each side will be recorded. The '30 s chair stand' test has been demonstrated to be a valid and reliable measure of proximal lower limb strength in older adults. The subject is asked to stand upright from a chair with arms folded across the chest, then to sit down again and then repeat the action at his/her own/fastest pace over a 30 s period. The final test score is the number of times that the subject rises to a full stand from the seated position with arms folded within 30 s [39]. To assess the strength of the m. quadriceps, patients will also be asked to perform three maximal voluntary knee extensions with the dominant leg at 60 degrees knee angle. The highest peak torque (Nm) of the three attempts will be used for analysis.

3. Fatigue

Two self-report questionnaires will be used to assess fatigue: the Multidimensional Fatigue Inventory (MFI) and the Fatigue Quality List (FQL). The MFI is a questionnaire consisting of 20 statements for which the person has to indicate on a 0-5 scale to what extent the particular statement applies to him or her. The statement refers to aspects of fatigue experienced during the previous days. Higher scores indicate a higher degree of fatigue. This self-report instrument consists of five subscales based on different dimensions: general fatigue, physical fatigue, reduced activity, reduced motivation and mental fatigue [40,41].

The patients' perception and appraisal of experienced fatigue will be assessed with the FQL [42]. The FQL consists of 25 adjectives describing the fatigue experience, organized into 4 subscales: frustrating, exhausting, pleasant, and frightening. An exploratory hypothesis is that, even if levels of fatigue severity do not change significantly over time, the subjective experience and perceived meaning of the fatigue may change as a function of physical exercise (e.g., from frustrating to pleasant, due to attributing the symptoms of fatigue to physical exertion rather than effects of the disease or its treatment). This necessitates the use of 2 questionnaires for measuring fatigue.

9.2 Secondary outcome measures:

1. Body composition and bone mineral density

Body composition and bone mineral density will be measured using whole body dual-energy X-ray (DXA). This method has been shown to give accurate and reproducible measurements of body composition [43]. The total radiation exposure is minimal. DXA scans will be performed at the T0 and T2 time point only. Body composition will also be estimated with the use of antrometry data (height, weight, waist and hip circumferences, and thickness of four skinfolds i.e. (biceps, triceps, suprailiacal and subscapular).

2. Health-related quality of life (HRQL)

The EORTC Core Quality of Life Questionnaire C30 (QLQ-C30) is a questionnaire developed to assess HRQL of cancer patients [44]. The EORTC QLQ-C30 encompasses 30 items divided in five functional scales (physical, role, emotional, cognitive, social), three symptom scales (pain, fatigue, and nausea and vomiting) and an overall QoL scale. Additional single items address other symptoms commonly experienced by cancer patients (e.g., insomnia, diarrhea, constipation, etc.). The EORTC myeloma module (QLQ-MY20) is used to receive extra information to assess HRQL in MM patients. This questionnaire measures for instance body image and future perspective and different aspects of pain [50]. Furthermore, neurotoxicity will be scored using the ZLZ-CIPN20 questionnaire, an EORTC quality of life questionnaire specifically developed to assess chemotherapy-induced peripheral neuropathy [45]. Neurotoxicity is an important side effect of chemotherapy used in this patient population (e.g. vincristin, bortezomib, cisplatin) and can hamper the ability to train these patients.
The EuroQol measures the health-related quality of life [46]. This is an important instrument for the purpose of economic evaluation (see paragraph 11.2.3).

3. Physical activity level

Physical activity will be measured with the PASE Questionnaire [47]. In addition physical activity will be assessed by the Actitrainer accelerometer (Actigraph, Fort Walton Beach Florida, USA). The Actigraph is able to measure accelerations from 0.05 to 2.00 G. These accelerations are scored in "counts" per minute that provide information about how long and how intensive a patient has been physically active. The epoch will be set at 60 seconds and the measurement period will include 5 days including at least one weekend day.

4. Mood disturbance

Mood disturbance will be assessed with the 14-item Hospital Anxiety and Depression Scale (HADS; [48]). The HADS assesses symptoms of mood disturbance, yielding separate scale scores for anxiety and depression, as well as a total score. The questionnaire has been extensively used in cancer patients and has been validated for use in the Dutch population [49].

5. Functioning in daily life

Functioning in daily life will be assessed with the Impact on Participation and Autonomy (IPA) Questionnaire [50]. The IPA Questionnaire consists of 32 items assessing perceived level of Participation and autonomy, organized into 5 domains: autonomy in the home, family role, autonomy outside of the home, social relations, and work and education.

6. Return to work

The following indicators of return to work will be measured: (1). Time to partial and to full RTW (meaning number of calendar days between end of treatment and first day at work), (2) time to full RTW corrected for partial RTW, (3) partial and full RTW rate at T=1 and T=2.
(4) Details on hours worked per week, nature of work, and return to a different job will also be recorded.

7. Satisfaction with the intervention

Satisfaction with the intervention will be assessed in the intervention arm only. For this purpose a satisfaction questionnaire is developed consisting of questions about: (1) the perceived efficacy of and satisfaction with the intervention program, (2) need for changes to the program, (3) the willingness to recommend the program to other patients undergoing high dose chemotherapy and SCT.
A short evaluation questionnaire will also be administered to health care providers.

9.3 Other measurements

1. Clinical data, disease status and treatment

Clinical data, including date of diagnosis, stage and subtype of disease, and treatment history will be obtained from the medical records. During the follow up-period data on disease status (response to treatment, progression or relapse) and data on any additional treatment will be retrieved from the medical records. At T0, T1 and T2, a complete blood count (haemoglobin, platelets, WBC) will be performed in patients for whom no recent (< 3 weeks) blood values are available.

2. Sociodemographic and clinical data

Sociodemographic data such as age, education, marital status, living situation, medication use (including alternative medications) and life style variables (e.g. smoking) will be obtained at baseline using a questionnaire.

3. Moderating variables

A series of questions will be used to assess a number of potential moderating variables: (1) pre-illness lifestyle, (2) current attitudes towards and beliefs about exercise in general, (3) exercising after stem cell transplantation in particular, (4) potential predictors of compliance with the exercise program.
These questions are adapted from measures developed by Courneya and colleagues for use in evaluating exercise in cancer survivors, and are based on health behaviour theories, in particular the Theory of Planned Behaviour [51]. They will be asked at baseline only.

4. Costs from a societal perspective

Costs will be measured from a societal perspective. The following are considered in this study:

- Health care costs: the costs of oncological care, general practice care and physiotherapy; additional visits to other health care providers, prescriptions of medication, professional home care and hospitalization.
- Patient and family costs: out-of-pocket expenses (e.g. travel expenses), costs for sports and sports equipment, and costs of paid and unpaid help.

- Costs due to loss of production (absenteeism for patients with paid jobs and hours of inactivity for patients without a paid job).

These data will be collected though retrospective cost questionnaires administered on a monthly basis during the period between T=0 and T=1, and once every 3 months in the period between T=1 and T=2. Health care utilization will be valued using Dutch cost prices [52].

5. Adverse Events

The grading of toxicity and adverse events will be done using the most recent version of the NCI Common Terminology Criteria for Adverse Events, CTCAE version 4. A complete document maybe downloaded from the following site:

http://ctep.cancer.gov/reporting/ctc.html

9.4 Monitoring of compliance and attrition

Compliance with the exercise program in the intervention arm will be assessed by self-report and by objective measures (e.g., attendance at and duration of the exercise sessions, exercise logs, target intensity). Reasons for noncompliance/nonattendance will be recorded.

Subjects can withdraw from the study at any time for any reason if they wish to do so without any consequences. When applicable, patients will be approached to ask for their reasons. The investigator can decide to withdraw a subject from the study for urgent medical reasons. Individual subjects will not be replaced after withdrawal. The principle of intention-to-treat will be applied in this study. Subjects who have withdrawn from treatment will be asked to participate in the follow-up measurements.

10. Safety reporting

10.1 Adverse event (AE)

An adverse event (AE) is any untoward medical occurrence in a patient or clinical study subject during protocol treatment. An AE does not necessarily have a causal relationship with the treatment. An AE can therefore be any unfavourable and unintended sign (including an abnormal laboratory finding), symptom, or disease temporally associated with the treatment whether or not related to the treatment.

10.2 Serious adverse event (SAE)

A serious adverse event is defined as any untoward medical occurrence that at any dose results in:

- death.
- a life-threatening event (i.e. the patient was at immediate risk of death at the time the reaction was observed).
- hospitalization or prolongation of hospitalization.
- significant / persistent disability.
- any other medically important condition (i.e. important adverse reactions that are not immediately life threatening or do not result in death or hospitalization but may jeopardize the patient or may require intervention to prevent one of the other outcomes listed above).

Note that ANY death, whether due to side effects of the treatment or due to progressive disease or due to other causes is considered as a serious adverse event.

10.2.1 Unexpected SAE

Unexpected serious adverse events are those SAE's of which the nature or severity is not consistent with information in the relevant source documents.

10.2 Protocol treatment period

The protocol treatment period is defined as the period from the baseline measurements (T0) until 30 days of the end of treatment (T1 or until the start of another systemic anti-cancer treatment off protocol, if earlier.

10.3 Reporting of serious adverse events

During protocol treatment all deaths, all SAE's that are life threatening and any unexpected SAE must be reported to the study coordinators by fax within 24 hours of the initial observation of the event, except hospitalizations for:

- a standard procedure for protocol therapy administration. Hospitalization or prolonged hospitalization for a complication of therapy administration will be reported as a serious adverse event.
- the administration of blood or platelet transfusion. Hospitalization or prolonged hospitalizationfor a complication of such transfusion remains a reportable serious adverse event.
- prolonged hospitalization for technical, practical, or social reasons, in absence of an adverse event.
- a procedure that is planned (i.e., planned prior to starting of treatment on study; must be documented in the source document and the CRF).

All details should be documented on the Serious Adverse Event and Death Report. In circumstances where it is not possible to submit a complete report an initial report may be made giving only the

mandatory information. Initial reports must be followed-up by a complete report within a further 14 calendar days. All SAE Reports must be dated and signed by the responsible clinical investigator or one of his/her authorized staff members. At any time after the protocol treatment period, unexpected serious adverse events that are considered to be at least suspected to be related to protocol treatment must also be reported to the study coordinators using the same procedure, within 48 hours after the SAE or death was known to the investigator. The investigator will decide whether the serious adverse event is related to the treatment (i.e. unrelated, unlikely, possible, probable, definitely and not assessable) and the decision will be recorded on the Serious Adverse Event and Death Report. The assessment of causality is made by the investigator using the following relationship description:

UNRELATED: There is no evidence of any causal relationship

UNLIKELY: There is little evidence to suggest there is a causal relationship (e.g. the event did not occur within a reasonable time after administration of the trial medication). There is another reasonable explanation for the event (e.g. the patient's clinical condition, other concomitant treatments).

POSSIBLE: There is some evidence to suggest a causal relationship (e.g. because the event occurs within a reasonable time after the assessments). However, the influence of other factors may have contributed to the event (e.g. the patient's clinical condition, other concomitant treatments).

PROBABLE: There is evidence to suggest a causal relationship and the influence of other factors is unlikely.

DEFINITELY: There is clear evidence to suggest a causal relationship and other possible contributing factors can be ruled out.

NOT ASSESSABLE: There is insufficient or incomplete evidence to make a clinical judgement of the causal relationship.

The study coordinators will forward all reports within one working day of receipt to the central data manager. The report of an SAE will be the signal for the data manager to ask the investigator to complete and send as soon as possible all relevant information for the involved patient with details of treatment and outcome. It is of utmost importance that all SAE's (including all deaths due to any cause) are reported in a timely fashion.

Patients without a report of an SAE are implicitly considered alive without SAE. This information will be used in monitoring the incidence of SAE's, the estimation of overall survival and safety monitoring. The study coordinator has responsibility for reporting such events to the Ethics Committee, which approved the study within the required timelines. The study coordinator will report to all applicable Health Authorities within required timelines.

11. Statistical considerations

11.1 Sample size and statistical power calculations

The sample size calculations were estimated for the primary outcomes for a two-sided α=0.05, and a power of 80%. We expect a between group difference of 7.5±7 ml/kg/min in VO2 max, of 0.2±0.1 kg in hand grip strength and of 3.5±4 in fatigue (MFI). We need between 25 and 42 subjects per group to detect these differences between the intervention and control group. Assuming a dropout rate of 30% (15% of the patients in our database who had undergone autologous SCT for MM< HL or NHL have an early relapse within 6 months, and are therefore expected not to complete the intervention program, and may in addition not be able to complete the study assessments. Furthermore, 15% may drop out because of other reasons) we need to enrol 60 subjects per group.
In the five participating transplant centers, yearly 180-210 autologous SCT are being performed. In 2007 170 patients received an autologous SCT for MM, HL or NHL (MM: 105 patients; NHL: 55 patients, HL: 10 patients). Taking in consideration all patients who will be ineligible for randomization (treatment related mortality SCT 4.3% = 3 patients; 10% of MM patients receiving an allogeneic SCT = 11 patients; 20% of MM patients having extensive osteolytic lesions with risk of fracture = 22 patients) we estimate that of the 170 patients transplanted yearly, 137 will be eligible, the majority of whom are expected to be interested in participating in the study (60 patients are needed on a yearly basis). This indicates that the proposed study is feasible with a projected inclusion time of 2 years.

11.2 Statistical analysis

11.2.1 Baseline characteristics

Differences in baseline characteristics between intervention and control groups will be tested using independent t-tests, Mann Whitney U tests and chi-square tests.

11.2.2 Primary analysis

Data will be analyzed on an intention-to-treat basis: patients who have been randomized to the intervention group but for whatever reason do not start or continue with training, will be encouraged to still undergo the assessments. Additionally, a per-protocol analysis will be performed with the patients who have completed at least 75% of the training sessions. Longitudinal regression analysis will be used to assess changes in each outcome measure. The two follow-up measurements will be defined as dependent variable and multi-level analysis with three levels will be used, 1) treatment center 2) time of follow-up measurement (values corresponding with performance at t=1 and t=2); 3) individual. Regression coefficients indicate differences between intervention and control group. Regression models will be adjusted for baseline values, age and gender. To test our hypothesis that the changes in physical fitness will mediate, in part, the beneficial effect of exercise on fatigue and quality of life, a mediation analysis will be performed using Latent variable structural equation modelling.
For *hypothesis 3* scores on the self-reported measures of mood state, physical activity and HRQL will be calculated according to existing scoring algorithms. Next multilevel longitudinal regression analyses will be conducted to assess differences in each outcome measure between intervention and control group at follow-up. Regression coefficients indicate differences between intervention and control groups. Regression models will be adjusted for gender, age and baseline values. For *hypothesis 4* (examining the differences in return to work), we will use Kaplan Meier's and the Cox proportional hazard regression for recurrent events. The general idea behind this analysis is that the different time periods are analysed separately adjusted for the fact that the time periods within one patient are dependent. Recurrences of sick leave for any reason during follow-up will be added to the Cox proportional hazards model with the

time to event approach, in which only the transitions from sick leave to return to work are taken into account.

11.2.3 Economic evaluation

This project will include an economic evaluation in collaboration with an expert in this field from the EMGO+ Institute for Health and Care Research. The aim of the economic evaluation will be to determine and compare the difference in total societal costs between the intervention and the control group and to relate these costs to the difference in effects between the two groups for the primary outcomes. The economic evaluation will be performed according to the intention-to-treat principle and from a societal perspective.

Both cost-effectiveness and cost-utility analyses will be performed. The cost-effectiveness ratios will be calculated by dividing the difference between the mean costs of the two treatment groups by the difference in the mean effects of the two treatment groups. This ratio will include the primary clinical effect measures of the trial. The cost-utility ratio will express the additional costs of the intervention per quality adjusted life year (QALY). Utilities will be measured using the EuroQol (EQ5D) at baseline, at the end of the treatment and at twelve months. The EuroQol is a health-related quality of life instrument that provides a single index of an individual's quality of life. It consists of 5 dimensions resulting in 243 possible health states [52]. The health states will be valued using Dutch tariffs.

Multiple Imputation (MI) using the Multivariate Imputation by Chained Equations (MICE) will be used to impute missing cost and effect data. Bootstrapping will be used to estimate confidence intervals around the mean differences in costs between the treatment groups. The uncertainty surrounding the cost-effectiveness and cost-utilities ratios will also be estimated using bootstrapping techniques. The bootstrapped cost-effect pairs will be used to estimate cost-effectiveness and cost-utility planes, and cost-effectiveness acceptability curves. Sensitivity analysis on the most important cost drivers will be performed in order to assess the robustness of the results.

12. Ethical and regulatory standards

12.1. Ethical principles

This protocol is in accordance with the principles laid down by the 18th world medical assembly (Helsinki, 1964) and amendments laid down by the 29th (Tokyo, 1975), the 35th (Venice, 1983) and the 41st (Hong Kong, 1989) World Medical Assemblies.

12.2. Laws and regulations

This protocol is in accordance with laws and regulations of the country in which the study is performed.

12.3. Informed Consent

The informed consent document will be used to explain in simple terms, before persons are entered into this study, the nature, scope and possible consequences of the study. The participant will give consent in writing. The signature of the physician and participant must confirm the participant's consent. The investigator is responsible to see that informed consent is obtained from the participant and for obtaining the appropriate signatures and dates on the informed consent document prior to the performance of any protocol procedure. The signed informed consent forms remain with the investigator.

12.4. Ethical review

Before start of the study, the study protocol and/or appropriate documents will be submitted to the ethical review committee (ERC), in accordance with local legal requirements. Only after approval will the study begin at the investigative site. The ERC will also be informed of all amendments and if necessary approval must be sought for ethical aspects.

12.5. Confidentiality

Personal information on the patients will be treated confidentially and anonymously according to the Wet Bescherming Persoongegevens. All patient names will be kept secret to anyone other than the investigator. Participants will be numbered consecutively in the order in which they are included in the study, the next participant receiving the next available number. The number allotted to them during the study will identify patients throughout documentation and evaluation. The participants will be told that all study findings will be stored on computer and handled in strictest confidence formulated in the Wet op de geneeskundige behandelovereenkomst (WGBO).

12.6 Compensation for injury

In accordance with Dutch law and the W.M.O., an insurance policy, covering all participating patients, has been effected as mentioned in the patient information form.

13. Administrative aspects and publication

13.1 Data monitoring

Data monitoring will be performed by a certified clinical research associate of our institute. The monitor will compare the data entered into the database with the hospital or clinic records (source documents). The nature and location of all source documents will be identified to ensure that all sources of original data required to complete the database are known to the investigational staff and are accessible for verification. At a minimum, source documentation must be available to substantiate: subject identification, eligibility and participation; proper informed consent procedures; dates of visits; adherence to protocol procedures; records of safety and efficacy parameters; adequate reporting and follow-up of adverse events; date of subject completion, discontinuation from treatment, or withdrawal from the study, and the reason if appropriate.
Direct access to source documentation (medical records) must be allowed for the purpose of verifying that the data recorded in the database are consistent with the original source data.

13.2 Annual progress report

The investigator will submit a summary of the progress of the trial to the accredited Medical Ethical Committee once a year. Information will be provided on the date of inclusion of the first subject, numbers of subjects included and numbers of subjects that have completed the trial, serious adverse events, other problems, and amendments.

13.3 End of study report

The investigator will notify the accredited Medical Ethical Committee of the end of the study within a period of 8 weeks. The end of the study is defined as the last patient's last visit.
In case the study is ended prematurely, the investigator will notify the accredited Medical Ethical Committee, including the reasons for the premature termination.
Within one year after the end of the study, the investigator will submit a final study report with the results of the study, including any publications/abstracts of the study, to the accredited Medical Ethical Committee.

13.4 Public disclosure and publication policy

It is our intention that the findings of the study be published in scientific journals and presented at scientific meetings. The responsibility for presentations and/or publications belongs to the investigators. Furthermore, a PHD thesis will be written and defended.

No restriction regarding the public disclosure and publication of the research data have been, or will be made by the funding agency.

14. Glossary of abbreviations

(in alphabetical order)

1-RM:	One repetition maximum
AE:	adverse event
AT:	Anaerobic threshold
CO_2	Carbon dioxide
DXA:	Dual energy X-ray
ECG:	Electrocardiogram
f max	maximal respiratory frequency
HDC:	High-dose chemotherapy
HL:	Hodgkin's lymphoma
HR:	Heart rate
HRmax:	maximal heart rate
HRQL:	Health-related quality of life
MET:	Metabolic unit
MM:	Multiple myeloma
MMC:	Maxima Medical Center
NHL	Non-Hodgkin's lymphoma
O_2	Oxygen
QUALY	Quality adjusted life year
RER:	Respiratory exchange ratio
RCT:	Randomized controlled trial
RPM:	revolutions per minute
RTW	Return to work
S	% saturation of Hb with O_2
SAE:	serious adverse event
SCT:	Stem cell transplantation
SUSAR:	Suspected unexpected serious adverse reaction
$VO_{2\ max:}$	Maximal aerobic power
$VO_{2:}$	Oxygen uptake
$VCO_{2:}$	Carbon dioxide output
V_{Emax}	maximal ventilation
WGBO:	'*Wet op de geneeskundige behandelovereenkomst*'
Wmax:	Maximal resistance
WMO:	'*Wet medisch-wetenschappelijk onderzoek*'

15. References

1. Andrykowski MA, Bishop MM, Hahn EA, Cella DF, Beaumont JL, Brady MJ *et al.*: **Long-term health-related quality of life, growth, and spiritual well-being after hematopoietic stem-cell transplantation.** *J Clin Oncol* 2005, **23:** 599-608.

2. Andrykowski MA, Greiner CB, Altmaier EM, Burish TG, Antin JH, Gingrich R *et al.*: **Quality of life following bone marrow transplantation: findings from a multicentre study.** *Br J Cancer* 1995, **71:** 1322-1329.

3. Syrjala KL, Chapko MK, Vitaliano PP, Cummings C, Sullivan KM: **Recovery after allogeneic marrow transplantation: prospective study of predictors of long-term physical and psychosocial functioning.** *Bone Marrow Transplant* 1993, **11:** 319-327.

4. Lucia A, Earnest C, Perez M: **Cancer-related fatigue: can exercise physiology assist oncologists?** *Lancet Oncol* 2003, **4:** 616-625.

5. Coleman EA, Coon S, Hall-Barrow J, Richards K, Gaylor D, Stewart B: **Feasibility of exercise during treatment for multiple myeloma.** *Cancer Nurs* 2003, **26:** 410-419.

6. Dimeo F, Bertz H, Finke J, Fetscher S, Mertelsmann R, Keul J: **An aerobic exercise program for patients with haematological malignancies after bone marrow transplantation.** *Bone Marrow Transplant* 1996, **18:** 1157-1160.

7. Dimeo F, Fetscher S, Lange W, Mertelsmann R, Keul J: **Effects of aerobic exercise on the physical performance and incidence of treatment-related complications after high-dose chemotherapy.** *Blood* 1997, **90:** 3390-3394.

8. Dimeo FC, Tilmann MH, Bertz H, Kanz L, Mertelsmann R, Keul J: **Aerobic exercise in the rehabilitation of cancer patients after high dose chemotherapy and autologous peripheral stem cell transplantation.** *Cancer* 1997, **79:** 1717-1722.

9. Dimeo FC, Stieglitz RD, Novelli-Fischer U, Fetscher S, Keul J: **Effects of physical activity on the fatigue and psychologic status of cancer patients during chemotherapy.** *Cancer* 1999, **85:** 2273-2277.

10. Hayes S, Davies PS, Parker T, Bashford J, Newman B: **Quality of life changes following peripheral blood stem cell transplantation and participation in a mixed-type, moderate-intensity, exercise program.** *Bone Marrow Transplant* 2004, **33:** 553-558.

11. Hayes SC, Davies PS, Parker TW, Bashford J, Green A: **Role of a mixed type, moderate intensity exercise programme after peripheral blood stem cell transplantation.** *Br J Sports Med* 2004, **38:** 304-309.

12. Wilson RW, Jacobsen PB, Fields KK: **Pilot study of a home-based aerobic exercise program for sedentary cancer survivors treated with hematopoietic stem cell transplantation.** *Bone Marrow Transplant* 2005, **35:** 721-727.

13. Liu RD, Chinapaw MJ, Huijgens PC, van MW: **Physical exercise interventions in haematological cancer patients, feasible to conduct but effectiveness to be established: a systematic literature review.** *Cancer Treat Rev* 2009, **35:** 185-192.

14. Wiskemann J, Huber G: **Physical exercise as adjuvant therapy for patients undergoing hematopoietic stem cell transplantation.** *Bone Marrow Transplant* 2008, **41:** 321-329.

15. Argiles JM, Busquets S, Felipe A, Lopez-Soriano FJ: **Molecular mechanisms involved in muscle wasting in cancer and ageing: cachexia versus sarcopenia.** *Int J Biochem Cell Biol* 2005, **37:** 1084-1104.

16. Watson T, Mock V: **Exercise as an intervention for cancer-related fatigue.** *Phys Ther* 2004, **84:** 736-743.

17. Tisdale MJ: **Cachexia in cancer patients.** *Nat Rev Cancer* 2002, **2:** 862-871.

18. Tisdale MJ: **Mechanisms of cancer cachexia.** *Physiol Rev* 2009, **89:** 381-410.

19. Brill PA, Macera CA, Davis DR, Blair SN, Gordon N: **Muscular strength and physical function.** *Med Sci Sports Exerc* 2000, **32:** 412-416.

20. Yarasheski KE, Zachwieja JJ, Bier DM: **Acute effects of resistance exercise on muscle protein synthesis rate in young and elderly men and women.** *Am J Physiol* 1993, **265:** E210-E214.

21. Cheema B, Gaul CA, Lane K, Fiatarone Singh MA: **Progressive resistance training in breast cancer: a systematic review of clinical trials.** *Breast Cancer Res Treat* 2008, **109:** 9-26.

22. Galvao DA, Newton RU: **Review of exercise intervention studies in cancer patients.** *J Clin Oncol* 2005, **23:** 899-909.

23. De Backer I, Van Breda E., Vreugdenhil A, Nijziel MR, Kester AD, Schep G: **High-intensity strength training improves quality of life in cancer survivors.** *Acta Oncol* 2007, **46:** 1143-1151.

24. De Backer I, Vreugdenhil G, Nijziel MR, Kester AD, Van Breda E., Schep G: **Long-term follow-up after cancer rehabilitation using high-intensity resistance training: persistent improvement of physical performance and quality of life.** *Br J Cancer* 2008, **99:** 30-36.

25. Freeman M, Vose J, Bennett C, Anderson J, Kessinger A, Turner K *et al.*: **Costs of care associated with high-dose therapy and autologous transplantation for non-Hodgkin's lymphoma: results from the University of Nebraska Medical Center 1989 to 1995.** *Bone Marrow Transplant* 1999, **24:** 679-684.

26. Ghatnekar O, Alvegard T, Conradi N, Lenhoff S, Mellqvist UH, Persson U *et al.*: **Direct hospital resource utilization and costs of treating patients with multiple myeloma in Southwest Sweden: a 5-year retrospective analysis.** *Clin Ther* 2008, **30:** 1704-1713.

27. Shankar SM, Carter A, Sun CL, Francisco L, Baker KS, Gurney JG *et al.*: **Health care utilization by adult long-term survivors of hematopoietic cell transplant: report from the Bone Marrow Transplant Survivor Study.** *Cancer Epidemiol Biomarkers Prev* 2007, **16:** 834-839.

28. Chao NJ, Tierney DK, Bloom JR, Long GD, Barr TA, Stallbaum BA *et al.*: **Dynamic assessment of quality of life after autologous bone marrow transplantation.** *Blood* 1992, **80:** 825-830.

29. Hensel M, Egerer G, Schneeweiss A, Goldschmidt H, Ho AD: **Quality of life and rehabilitation in social and professional life after autologous stem cell transplantation.** *Ann Oncol* 2002, **13:** 209-217.

30. Lee SJ, Fairclough D, Parsons SK, Soiffer RJ, Fisher DC, Schlossman RL *et al.*: **Recovery after stem-cell transplantation for hematologic diseases.** *J Clin Oncol* 2001, **19:** 242-252.

31. Wong FL, Francisco L, Togawa K, Bosworth A, Gonzales M, Hanby C *et al.*: **Long-term recovery after hematopoietic cell transplantation: predictors of quality-of-life concerns.** *Blood* 2010, **115:** 2508-2519.

32. TENALEA Clinical Trial Data Management System. An online, central randomisation service, currently in deployment phase with a grant from the e-TEN programme of the European Union (LSHC-CT-510736). 2010.
Ref Type: Computer Program

33. Haskell WL, Lee IM, Pate RR, Powell KE, Blair SN, Franklin BA *et al.*: **Physical activity and public health: updated recommendation for adults from the American College of Sports Medicine and the American Heart Association.** *Circulation* 2007, **116:** 1081-1093.

34. Borg GA: **Psychophysical bases of perceived exertion.** *Med Sci Sports Exerc* 1982, **14:** 377-381.

35. Korstjens I, Mesters I, van der Peet E, Gijsen B, van den Borne B: **Quality of life of cancer survivors after physical and psychosocial rehabilitation.** *Eur J Cancer Prev* 2006, **15:** 541-547.

36. Korstjens I, Mesters I, Gijsen B, van den Borne B: **Cancer patients' view on rehabilitation and quality of life: a programme audit.** *Eur J Cancer Care (Engl)* 2008, **17:** 290-297.

37. ERS Task Force on Standardization of Clinical Exercise Testing.: **Clinical exercise testing with reference to lung diseases: indications, standardization and interpretation strategies. European Respiratory Society.** *Eur Respir J* 1997, **10:** 2662-2689.

38. Wasserman K, Hansen JE, Sue DY, Stringer WW, Whipp B.J.: *Principles of Exercise Testing and Interpretation. Including Pathophysiology and Clinical Applications*, fourth edn. Philadelphia: Lippincott Williams & Wilkins; 2005.

39. Rikli RE, Jones CE: **Development and validation of a functional fitness test for community-residing older adults.** *Journal of aging and physical activity* 1999, **7:** 129-161.

40. Echteld MA, Passchier J, Teunissen S, Claessen S, de WR, van der Rijt CC: **Multidimensional fatigue and its correlates in hospitalised advanced cancer patients.** *Eur J Cancer* 2007, **43:** 1030-1036.

41. Smets EM, Garssen B, Bonke B, De Haes JC: **The Multidimensional Fatigue Inventory (MFI) psychometric qualities of an instrument to assess fatigue.** *J Psychosom Res* 1995, **39:** 315-325.

42. Gielissen MF, Knoop H, Servaes P, Kalkman JS, Huibers MJ, Verhagen S *et al.*: **Differences in the experience of fatigue in patients and healthy controls: patients' descriptions.** *Health Qual Life Outcomes* 2007, **5:** 36.

43. Aasen G, Fagertun H, Halse J: **Body composition analysis by dual X-ray absorptiometry: in vivo and in vitro comparison of three different fan-beam instruments.** *Scand J Clin Lab Invest* 2006, **66:** 659-666.

44. Aaronson NK, Ahmedzai S, Bergman B, Bullinger M, Cull A, Duez NJ *et al.*: **The European Organization for Research and Treatment of Cancer QLQ-C30: a quality-of-life instrument for use in international clinical trials in oncology.** *J Natl Cancer Inst* 1993, **85:** 365-376.

45. Postma TJ, Aaronson NK, Heimans JJ, Muller MJ, Hildebrand JG, Delattre JY *et al.*: **The development of an EORTC quality of life questionnaire to assess chemotherapy-induced peripheral neuropathy: the QLQ-CIPN20.** *Eur J Cancer* 2005, **41:** 1135-1139.

46. Kopec JA, Willison KD: **A comparative review of four preference-weighted measures of health-related quality of life.** *J Clin Epidemiol* 2003, **56:** 317-325.

47. Washburn RA, Smith KW, Jette AM, Janney CA: **The Physical Activity Scale for the Elderly (PASE): development and evaluation.** *J Clin Epidemiol* 1993, **46:** 153-162.

48. Zigmond AS, Snaith RP: **The hospital anxiety and depression scale.** *Acta Psychiatr Scand* 1983, **67:** 361-370.

49. Spinhoven P, Ormel J, Sloekers PP, Kempen GI, Speckens AE, van Hemert AM: **A validation study of the Hospital Anxiety and Depression Scale (HADS) in different groups of Dutch subjects.** *Psychol Med* 1997, **27:** 363-370.

50. Cardol M, Beelen A, van den Bos GA, de Jong BA, de G, I, de Haan RJ: **Responsiveness of the Impact on Participation and Autonomy questionnaire.** *Arch Phys Med Rehabil* 2002, **83:** 1524-1529.

51. Courneya KS, Friedenreich CM: **Utility of the theory of planned behavior for understanding exercise during breast cancer treatment.** *Psychooncology* 1999, **8:** 112-122.

52. OOSTENBRINK JB, BOUWMANS CAM, KOOPMANSCHAP MA, RUTTEN FFH: **Handleiding voor kostenonderzoek, methoden en standaard kostprijzen voor economische evaluaties in de gezondheidzorg.** *Geactualiseerde versie* 2004.
