# Supplementary figures and images for: Randomized controlled trial on the effects of a supervised high intensity exercise program in patients with a hematologic malignancy treated with autologous stem cell transplantation: Results from the EXIST study
Source: PLoS One. 2017 Jul 20;12(7):e0181313. doi: 10.1371/journal.pone.0181313 (PMC5519072; doi:10.1371/journal.pone.0181313)

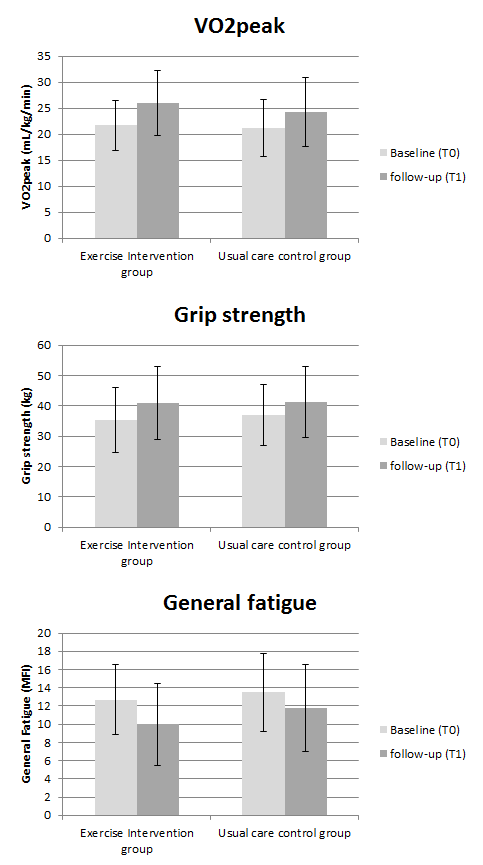

Supplement: S1 Fig — (TIF) [file pone.0181313.s003.tif]
